# Non-classical monocytes are biased progenitors of wound healing macrophages during soft tissue injury

**DOI:** 10.1038/s41598-017-00477-1

**Published:** 2017-03-27

**Authors:** Claire E. Olingy, Cheryl L. San Emeterio, Molly E. Ogle, Jack R. Krieger, Anthony C. Bruce, David D. Pfau, Brett T. Jordan, Shayn M. Peirce, Edward A. Botchwey

**Affiliations:** 1 Wallace H. Coulter Department of Biomedical Engineering, Georgia Institute of Technology and Emory University, 315 Ferst Drive, Atlanta, GA 30332 USA; 2 Department of Biomedical Engineering, University of Virginia, Box 800759, Charlottesville, VA 22908 USA

## Abstract

Successful tissue repair requires the activities of myeloid cells such as monocytes and macrophages that guide the progression of inflammation and healing outcome. Immunoregenerative materials leverage the function of endogenous immune cells to orchestrate complex mechanisms of repair; however, a deeper understanding of innate immune cell function in inflamed tissues and their subsequent interactions with implanted materials is necessary to guide the design of these materials. Blood monocytes exist in two primary subpopulations, characterized as classical inflammatory or non-classical. While classical monocytes extravasate into inflamed tissue and give rise to macrophages or dendritic cells, the recruitment kinetics and functional role of non-classical monocytes remains unclear. Here, we demonstrate that circulating non-classical monocytes are directly recruited to polymer films within skin injuries, where they home to a perivascular niche and generate alternatively activated, wound healing macrophages. Selective labeling of blood monocyte subsets indicates that non-classical monocytes are biased progenitors of alternatively activated macrophages. On-site delivery of the immunomodulatory small molecule FTY720 recruits S1PR3-expressing non-classical monocytes that support vascular remodeling after injury. These results elucidate a previously unknown role for blood-derived non-classical monocytes as contributors to alternatively activated macrophages, highlighting them as key regulators of inflammatory response and regenerative outcome.

## Introduction

The mononuclear phagocyte system plays a multi-faceted role in maintaining tissue homeostasis and responding to pathological processes such as autoimmune diseases, cancer, and aberrant wound healing. Monocytes circulate in the bloodstream during steady state and are robustly recruited to sites of inflammation, where they exert functions that include clearance of cellular debris, promotion of angiogenesis, and restoration of tissue integrity^1^. The ontogeny of macrophages varies in different tissues, such that some tissue resident macrophages are seeded embryonically and self-renew in a similar manner to stem cells, whereas other macrophages (such as in the dermis or gut) are continually replenished by blood-derived monocytes^2–6^. Consequently, circulating blood monocytes are considered a highly plastic and dynamic system of innate immune cells that initiate processes of organ and tissue remodeling^1, 7, 8^. Immunologically smart interventions that exploit the division of labor between different monocyte and macrophage populations require an understanding of the roles that these cells play in promoting repair, as unchecked activity of innate immune cells can perpetuate tissue damage through chronic inflammation and fibrosis.

Two distinct subpopulations of monocytes have been identified in mouse and human blood, which can be distinguished by well-characterized surface protein expression profiles. Classical inflammatory monocytes are identified by Ly6C^hi^CX3CR1^lo^CD43^lo^ expression in mice (CD14^hi^CD16^−^ in human), whereas non-classical alternative monocytes are Ly6C^lo^CX3CR1^hi^CD43^hi^ in mice (CD14^+^CD16^+^ in human)^2^. A third population of intermediate monocytes characterized by intermediate expression of Ly6C in mice (CD14^hi^CD16^+^ in humans) are thought to complement the functions of non-classical monocytes and may preferentially differentiate into dendritic cells within inflamed tissues^9, 10^. Under homeostasis, classical monocytes in blood decrease Ly6C expression and become non-classical Ly6C^lo^ monocytes^5, 11^, which patrol the luminal side of resting endothelium^12^. Classical monocytes also survey steady-state tissues and can traffic to lymph nodes without differentiating into macrophages^13^. During inflammation, monocytes exit peripheral blood and extravasate into tissue, where they may transiently persist as monocytes without differentiation and exert a host of functions within the damaged tissue^13–17^. Classical inflammatory monocytes present in the acute phases of injury secrete pro-inflammatory cytokines such as IL-6, iNOS, and TNFα^17^ and exhibit high levels of matrix metalloproteinase and cathepsin production^18^. Conversely, Ly6C^lo^ monocytes present later during inflammation secrete high levels of vascular endothelial growth factor (VEGF) and IL-10 and can induce endothelial cell proliferation to promote arteriogenesis^14, 18, 19^. We have previously shown that strategies that enhance the early recruitment of Ly6C^lo^ monocytes couple with later increases in arteriolar expansion and angiogenic activity^20, 21^. Recruited monocytes can differentiate into macrophages, serving as an alternative source of wound macrophages to those derived from *in situ* proliferation of tissue resident populations^22^.

Macrophages are highly responsive to cues within the injury niche, enabling them to dynamically modify their behavior in response to changes in the microenvironment and display extremely varied phenotypes. Classically activated (“M1”) macrophages are primary players in pathogen destruction, secretion of inflammatory cytokines, and driving Th1-type responses^23^. Conversely, alternatively activated wound healing (“M2”) macrophages (of which a number of subtypes have been described^23^) are associated with pro-regenerative activities such as angiogenesis^24, 25^, extracellular matrix remodeling^26^, secretion of anti-inflammatory cytokines^23^, and resolution of inflammation^27^. The highly complex and heterogeneous nature of inflamed tissue microenvironments has rendered a general description of macrophage origin and function challenging. Within toxin-induced muscle injury^19^, liver fibrosis^28^, infection^29^, and autoimmune disease^30^, classical Ly6C^hi^ monocytes are recruited from circulation and undergo *in situ* differentiation to be the primary contributors of injury Ly6C^lo^ monocytes/macrophages. In contrast, sequential recruitment of classical Ly6C^hi^ followed by non-classical Ly6C^lo^ monocyte subsets after myocardial infarction^18^, and direct recruitment of adoptively transferred Ly6C^lo^ monocytes within excisional skin injury^20^ and during the development of inflammatory arthritis^31^ have been reported. However, whether specific populations of blood monocytes give rise to defined macrophage phenotypes surrounding implanted materials remains unknown.

Harnessing myeloid cell functions for regenerative medicine applications requires an understanding of the cues that direct the localization and fate of these cells. Immunoregenerative materials seek to leverage the function of endogenous immune cells to guide the progression of inflammation and repair damaged tissue^32^. For example, local delivery of stromal-derived factor-1 (SDF-1) from desulfated heparin-containing poly(ethylene glycol) (PEG) hydrogels increases the frequency of CXCR4^hi^Ly6C^lo^ monocytes, which promotes capillary network expansion^21^. Moreover, co-delivery of macrophage colony-stimulating factor (M-CSF) with VEGF from PEG hydrogels increases the density and maturity of corneal blood vessels compared to VEGF alone^33^. Recently, our lab demonstrated that poly(lactic-*co*-glycolic acid) (PLGA)-based delivery of the small molecule FTY720, an agonist of sphingosine-1-phosphate receptor (S1PR) 3, recruits non-classical monocytes to inflamed tissues and promotes arteriogenesis^20^. In the present study, we utilize cell labeling strategies to selectively track the fate of either classical Ly6C^hi^ or non-classical Ly6C^lo^ monocytes in response to biomaterial implantation within cutaneous wounds. We demonstrate that classical monocytes are able to give rise to both CD206− and CD206+ macrophages following monocyte depletion with clodronate liposomes, but labeled non-classical monocytes preferentially give rise to CD206+ M2-like macrophages. On-site delivery of the immunomodulatory small molecule FTY720 induces homing of extravasated non-classical monocytes to peri-implant vasculature. Subsequently, FTY720 promotes *in situ* generation of wound healing macrophages and vascular remodeling within ischemic skeletal muscle. These results shed light on the fate of specific monocyte populations following biomaterial implantation after injury and indicate that non-classical monocytes are a promising therapeutic target for harnessing pro-regenerative inflammation to promote repair.

## Results

### Skin wounding and biomaterial implantation induces monocyte trafficking

**Figure 1:**
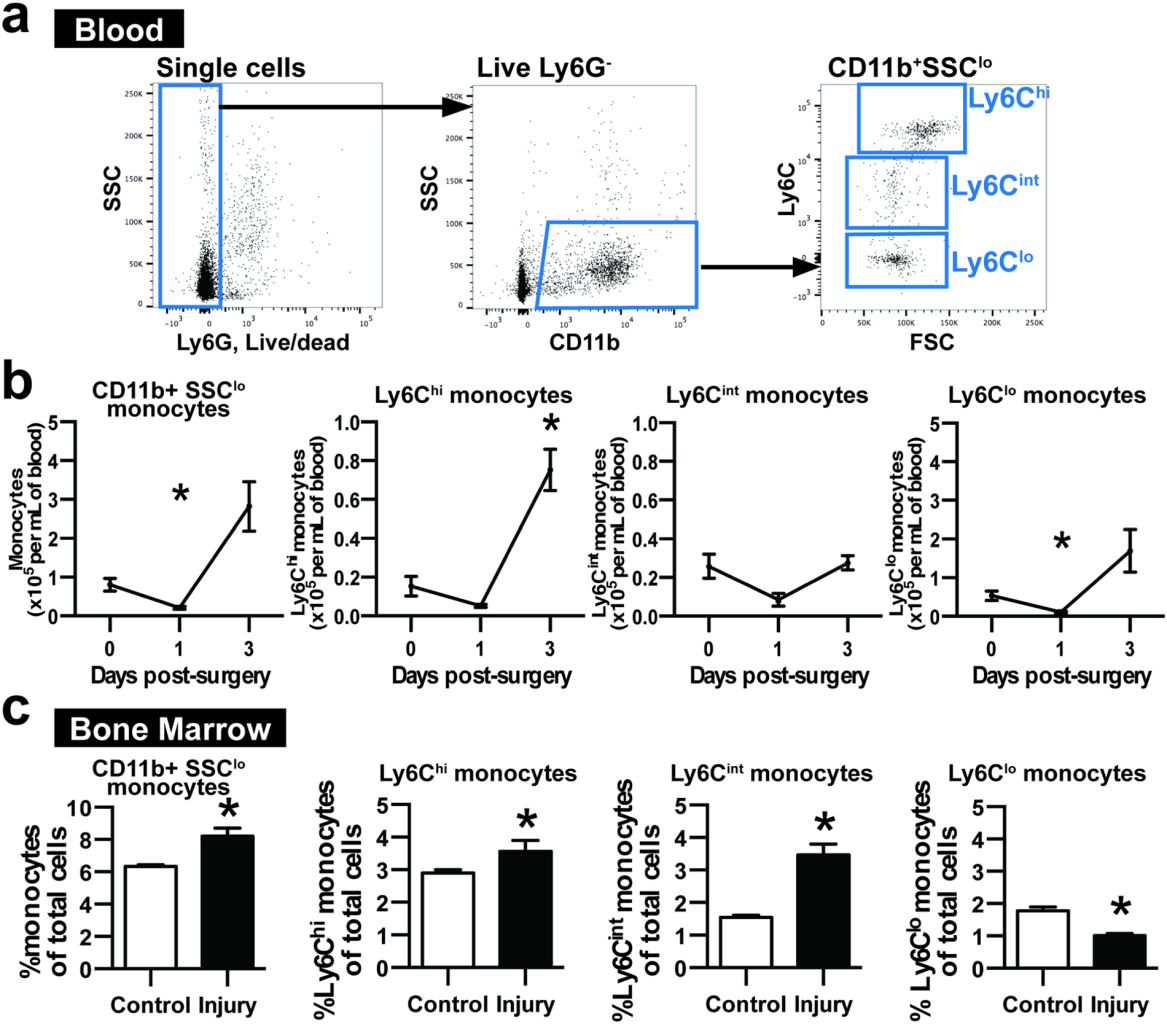
Wounding and biomaterial implantation induces acute changes in circulating monocytes. (**a**) White blood cells were gated on CD11b+SSC^lo^ expression and categorized as Ly6C^hi^, Ly6C^int^, and Ly6C^lo^ monocytes based on Ly6C expression. Ly6G+, dead white blood cells were excluded from analysis. (**b**) CD11b+SSC^lo^ monocytes and Ly6C^hi^, Ly6C^int^, and Ly6C^lo^ monocytes per milliliter of blood 1 and 3 days post-injury. *p < 0.05 relative to Day 0 blood, n = 5 animals per group. (**c**) Frequency of CD11b+SSC^lo^ monocytes and Ly6C^hi^, Ly6C^int^, and Ly6C^lo^ monocytes within bone marrow 3 days post-injury. *p < 0.05, n = 4–10 animals per group. Data presented as mean ± S.E.M. Statistical analyses performed using either two-way ANOVA with multiple comparisons or two-tailed t-tests.

The dorsal skinfold window chamber (DWC) model is a partial thickness excisional skin injury model that involves removing the epidermis and dermis to reveal the underlying sub-reticular vasculature. We have previously used this model to investigate the recruitment of distinct monocyte subsets to inflamed tissue surrounding biomaterial implants^20, 21, 34^. In this study, we tracked the fate of monocytes that are recruited in response to cutaneous wounding and material implantation. DWC surgery and implantation of a polymeric poly(lactic-*co*-glycolic acid) (PLGA) thin film 1mm in diameter decreased the frequency of blood monocytes by nearly four-fold 1 day post-injury, followed by a three-fold elevation by 3 days post-injury compared to blood taken at day 0 prior to surgery (Fig. 1a,b). Monocytes were identified based on CD11b+SSC^lo^Gr-1^lo^ expression, as Gr-1^hi^Ly6C^int^ cells are SSC^hi^ granulocytes (Supplemental Figure S1a). A three-fold decrease in the frequency of circulating classical Ly6C^hi^ monocytes was observed in the first day post-injury, followed by a five-fold increase by day 3 (Fig. 1b). Non-classical Ly6C^lo^ monocytes also initially decreased (by five-fold), but then increased by five-fold relative to day 0 (Fig. 1b) and were the most abundant type of monocyte in blood for the duration of the study (Supplemental Figure S1b). Ly6C^int^ monocytes were similarly decreased at day 1 by three-fold fold following injury but returned to baseline levels by day 3. These changes in circulating myeloid cell populations were accompanied by corresponding relative changes in bone marrow cell populations. At 3 days post-injury, higher frequencies of total, Ly6C^hi^, and Ly6C^int^ monocytes were detected in bone marrow, while the frequency of Ly6C^lo^ monocytes decreased (Fig. 1c). To further probe the fate of blood myeloid populations that were altered after injury, we utilized *in situ* labeling techniques to track blood monocytes.

### Injured skin recruits Ly6C^lo^ monocytes that give rise to CD206+ wound macrophages

**Figure 2:**
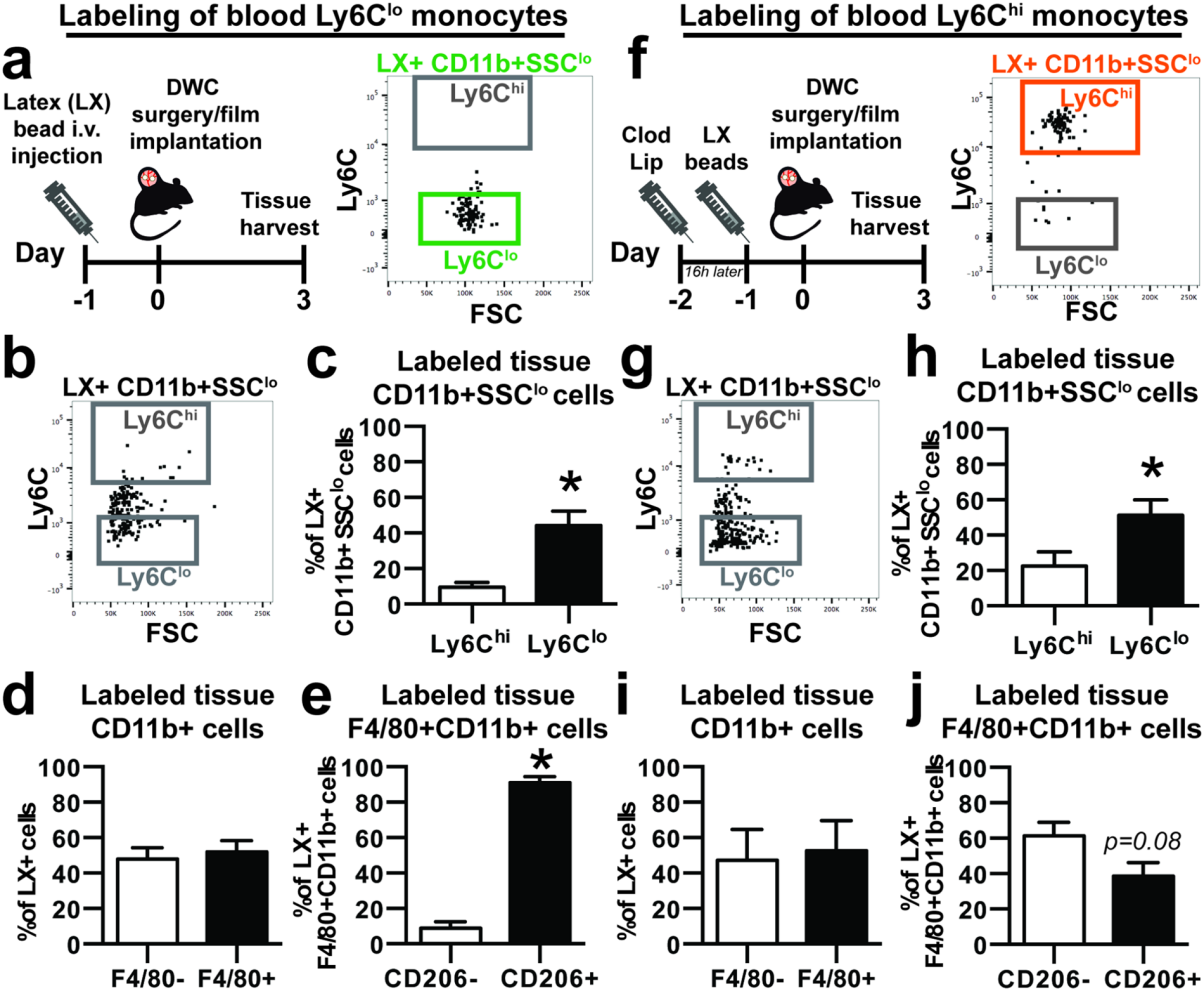
Circulating non-classical Ly6C^lo^ monocytes selectively give rise to CD206+ wound repair macrophages. (**a**) Latex (LX) beads were injected 1 day prior to DWC surgery to selectively label blood Ly6C^lo^ monocytes. Right, flow cytometry dot plot of labeled blood LX+CD11b+SSC^lo^ cells shows selective Ly6C^lo^ monocyte labeling 1 day post-injury. (**b,c**) Labeled monocytes collected from digested dorsal tissue 3 days post-injury. (**d**) Frequency of bead-labeled F4/80+ cells in animals with labeled blood Ly6C^lo^ monocytes. (**e**) Frequency of bead-labeled CD206+ cells out of total F4/80+CD11b+ cells. (**f**) Blood Ly6C^hi^ monocytes were selectively labeled by first depleting blood monocytes with i.v. clodronate liposome (Clod Lip) administration 2 days prior to injury, followed by LX bead-based labeling 16 h later. Right, flow cytometry dot plot of labeled blood LX+CD11b+SSC^lo^ cells shows selective Ly6C^hi^ monocyte labeling 1 day post-injury. (**g**,**h**) Labeled monocytes collected from digested dorsal tissue 3 days post-injury. (**i**) Frequency of bead-labeled F4/80+ cells in animals with labeled blood Ly6C^hi^ monocytes. (**j**) Frequency of bead-labeled CD206+ cells out of total F4/80+CD11b+ cells. Data presented as mean ± S.E.M. Statistical analyses performed using two-tailed t-tests. *p < 0.05, n = 4–11 animals per group.

Circulating monocyte subsets were labeled *in vivo* prior to DWC surgery to facilitate cell tracking of each monocyte population as they entered inflamed dorsal tissue surrounding polymer implants. Non-classical Ly6C^lo^ monocyte labeling was performed by intravenous administration of fluorescent latex beads (Fig. 2a), as previously described^35, 36^. Within two hours of intravascular administration, latex beads are phagocytosed and equally distribute within Ly6C^lo^ and Ly6C^hi^ blood monocyte populations. However, by 24 hours after injection, latex beads primarily label Ly6C^lo^ monocytes due to physiological conversion of labeled Ly6C^hi^ monocytes and this labeling is sustained for up to 1 week^35^. Utilizing this strategy, blood Ly6C^lo^ monocytes were selectively labeled compared to classical Ly6C^hi^ monocytes after DWC (83.4 ± 9.7% Ly6C^lo^ monocytes vs. 14.5 ± 9.3% Ly6C^int^ monocytes and 0.0 ± 0.0% Ly6C^hi^ monocytes, day 1 post-injury) and the label was retained at similar proportions for the duration of the study (Fig. 2a, Supplemental Figure S1c,d). Analysis of digested explanted dorsal skin tissue (Supplemental Figure S2a,b) showed labeled cells originating from Ly6C^lo^ monocytes primarily remained Ly6C^lo^ within tissue. The lower frequency of Ly6C^hi^ monocytes carrying the label in the tissue (9.6 ± 2.6% of LX+CD11b+SSC^lo^ cells) compared to the frequency of labeled Ly6C^lo^ cells (44.3 ± 8.2% of LX+CD11b+SSC^lo^ cells) indicates that circulating Ly6C^lo^ monocytes do not become Ly6C^hi^ post-extravasation (Fig. 2b,c). Approximately half of labeled cells expressed F4/80, indicating that around half of recruited Ly6C^lo^ monocytes convert into macrophages or are phagocytosed by macrophages by 3 days post-injury (Fig. 2d). About half of total F4/80+CD11b+ cells expressed CD206 (Supplemental Figure S2c), whereas labeled cells were more likely to be immunophenotyped as CD206+ macrophages (91.0 ± 3.4% of LX+F4/80+CD11b+ cells) than CD206− macrophages (Fig. 2e). These data suggest that blood-derived Ly6C^lo^ monocytes preferentially give rise to CD206+ wound macrophages in inflamed tissue surrounding material implants.

We then tracked circulating Ly6C^hi^ blood monocytes to explore whether these cells adopted similar fates during inflammation. Ly6C^hi^ monocytes were labeled by sequential administration of clodronate-loaded liposomes 2 days prior to injury followed by latex beads 16 hours later (Fig. 2f), as previously described^35, 36^. Clodronate liposomes administered intravascularly transiently deplete all blood monocytes, resulting in accumulation of latex beads in bone marrow cells that reappear in circulating Ly6C^hi^ monocytes 2 days later^35^. Pre-administration of clodronate liposomes before latex bead injection preferentially labeled Ly6C^hi^ monocytes (70.8 ± 13.3% Ly6C^hi^ monocytes vs. 26.9 ± 12.9% Ly6C^int^ monocytes and 2.7 ± 0.9% Ly6C^lo^ monocytes, day 1 post-surgery) for the duration of the study (Fig. 2f, Supplemental Figure S1e,f). A relatively low frequency of labeled Ly6C^hi^ monocytes was detected in digested tissue (Fig. 2g,h), confirming previous reports^14, 15, 19^ that recruited Ly6C^hi^ monocytes do not persist as Ly6C^hi^ monocytes post-extravasation. Ly6C^hi^ monocytes likely rapidly convert *in situ* into Ly6C^lo^ monocytes, as a significantly greater frequency of labeled Ly6C^lo^ monocytes than Ly6C^hi^ monocytes were detected (Fig. 2h). The frequency of F4/80+ cells within the latex bead-positive (LX+) population was not different between Ly6C^hi^ monocyte and Ly6C^lo^ monocyte labeling (47.5 ± 17.3% vs. 48.0 ± 6.2% of LX+ cells), suggesting that both monocyte populations are equally capable of acquiring a macrophage phenotype after extravasation (Fig. 2d,i). Labeled Ly6C^hi^ monocytes showed no preference for acquiring CD206 expression within 3 days of injury (Fig. 2j), indicating that although blood-derived Ly6C^hi^ monocytes can contribute to CD206+ macrophages after clodronate liposome administration, they do so at a lower frequency than Ly6C^lo^ monocytes.

### Reduction of circulating Ly6C^lo^ monocytes impairs CD206+ macrophage generation

**Figure 3:**
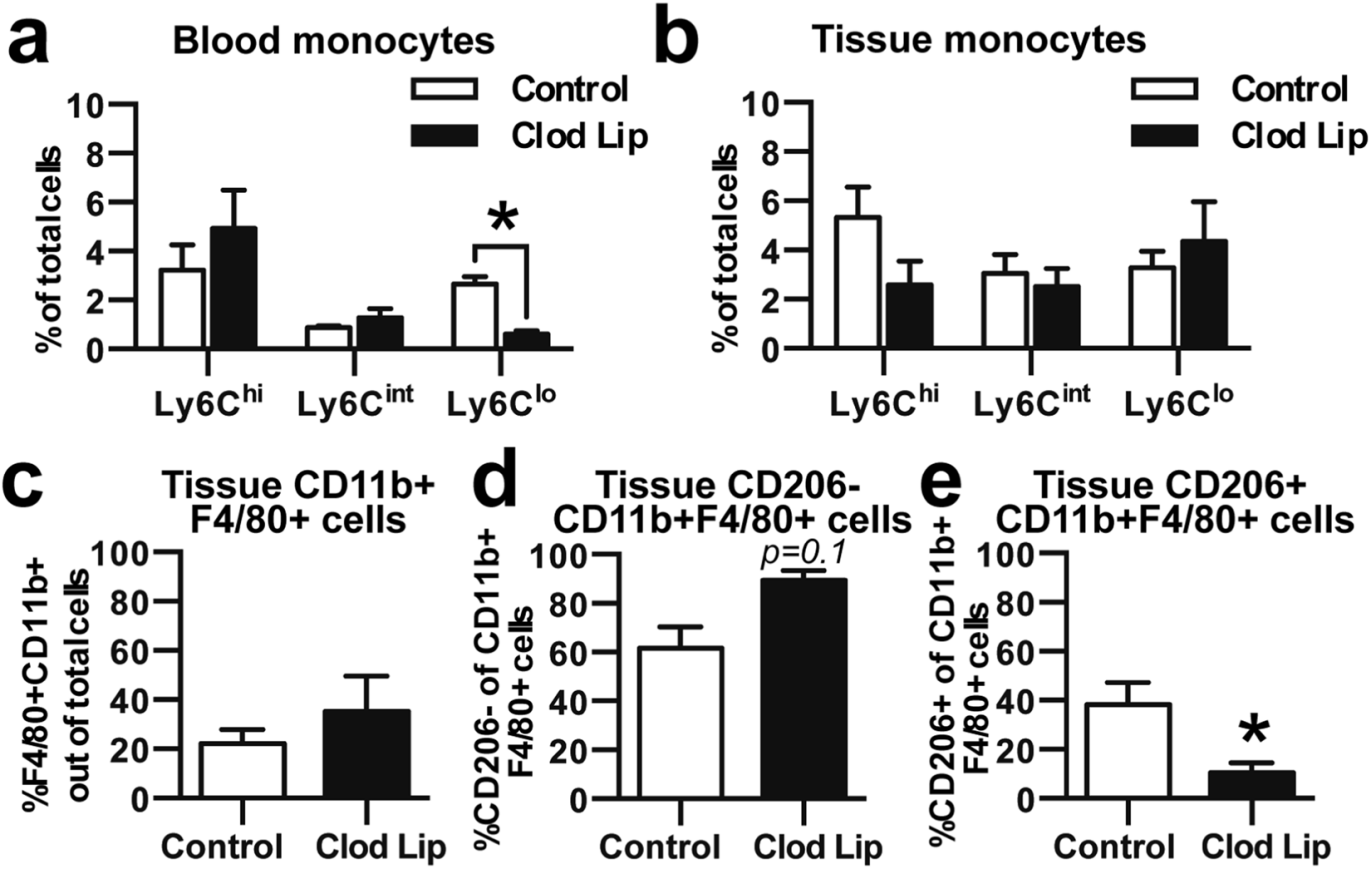
Loss of Ly6C^lo^ monocytes impairs generation of CD206+ alternatively activated macrophages. (**a**) Clodronate liposome (Clod Lip) administration two days before DWC surgery reduced the frequency in blood of Ly6C^lo^ monocytes, but not Ly6C^hi^ or Ly6C^int^ monocytes. (**b**) Frequency of Ly6C^lo^, Ly6C^int^, Ly6C^hi^ monocytes after Clod Lip administration in digested dorsal tissue 3 days post-injury. (**c**) Total CD11b+F4/80+ macrophages, (**d**) CD206− macrophages, and (**e**) CD206+ alternatively activated macrophages 3 days post-injury. Data presented as mean ± S.E.M. Statistical analyses were performed using two-tailed t-tests *p < 0.05, n = 4–8 animals per group.

Intravascular administration of clodronate liposomes transiently depletes all circulating monocytes; however, because blood Ly6C^lo^ monocytes are primarily derived from the conversion of Ly6C^hi^ monocytes, there is a delay in the repopulation of circulating Ly6C^lo^ monocytes^5, 11^. We employed this tool to examine how decreasing the quantity of circulating non-classical monocytes impacts the generation of CD206+ macrophages during wound healing. As expected, a deficit in blood Ly6C^lo^ monocytes, but not Ly6C^hi^ monocytes, was observed 5 days after clodronate administration (Fig. 3a). No differences in either monocyte subtype (Fig. 3b), total F4/80+ macrophages (Fig. 3c), or CD206− macrophages (Fig. 3d) were observed in digested tissue 3 days post-injury; however, a lower frequency of CD206+ macrophages was observed (Fig. 3e). These findings suggest that circulating Ly6C^lo^ monocytes are likely major contributors to the population of CD206+ wound macrophages.

### Adoptively transferred Ly6C^lo^ monocytes preferentially differentiate into CD301b+CD206+ macrophages

**Figure 4:**
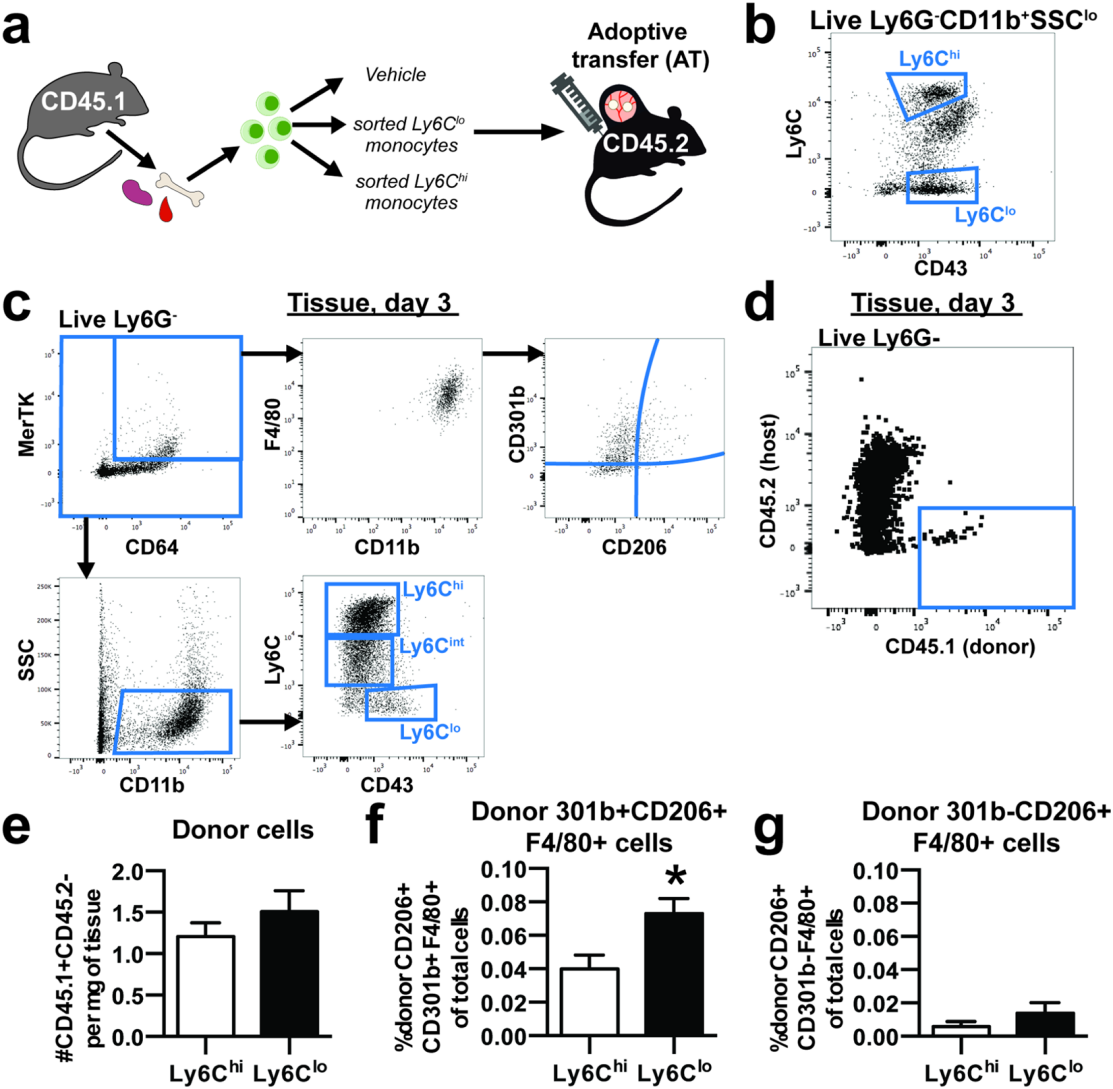
Adoptively transferred non-classical Ly6C^lo^ monocytes differentiate into CD206+ macrophages within inflamed peri-implant tissue. (**a**) CD45.1+ Ly6C^hi^ or Ly6C^lo^ monocytes sorted from pooled bone marrow, blood, and spleen were adoptively transferred by i.v. injection to CD45.2+ mice at the time of DWC surgery. (**b**) Monocytes were sorted based on CD11b+SSC^lo^Ly6G-expression and Ly6C^hi^CD43^lo^ expression or Ly6C^lo^CD43^hi^ expression. (**c**) Macrophage subpopulations in dorsal tissue at 3 days post injury were immunophenotyped as MerTK+CD64+ or F4/80+CD11b+ and characterized for CD301b and CD206 surface expression. Monocyte subpopulations in dorsal tissue were immunophenotyped as CD11b+SSC^lo^ and characterized for Ly6C and CD43 surface expression. (**d**) Donor-derived cells were identified in dorsal tissue 3 days post-injury as CD45.1+CD45.2−. (**e**) Total donor cells per milligram of tissue in mice receiving Ly6C^hi^ or Ly6C^lo^ monocytes. (**f**) Frequency of donor-derived CD301b+CD206+ macrophages (CD11b+F4/80+) in mice receiving adoptive transfer Ly6C^lo^ monocytes compared to mice receiving Ly6C^hi^ monocytes. (**g**) Frequency of donor-derived CD301b−CD206+ macrophages (CD11b+F4/80+). Data presented as mean ± S.E.M. Statistical analyses were performed using two-tailed t-tests *p < 0.05, n = 5–6 animals per group.

To complement *in vivo* bead-based labeling strategies and further investigate the role that blood monocytes play in macrophage generation during inflammation, we adoptively transferred sorted CD45.1+ Ly6C^hi^CD43^lo^ or Ly6C^lo^CD43^hi^ monocytes into CD45.2 mice at the time of DWC surgery (Fig. 4a,b). While Ly6C^lo^CD43^hi^ are a slightly more restricted population of Ly6C^lo^ monocytes, these two populations primarily overlap and the same is true for Ly6C^hi^CD43^lo^ monocytes (Supplemental Figure S2d). At 3 days post-injury, we assayed donor cells for expression of monocyte and macrophage markers (Fig. 4c, Supplemental Figure S3). A very low frequency of CD45.1+CD45.2− cells were detected in digested dorsal tissue (Fig. 4d, Supplemental Figure S4a,b), indicating that few donor cells are present 3 days post-injury. We detected a modest, but insignificant increase in the number of total donor cells (normalized to tissue mass) originating as Ly6C^lo^CD43^hi^ relative to those adoptively transferred as Ly6C^hi^CD43^lo^ (Fig. 4e). CD301b marks a population of macrophages that appear in the midphase of skin wound healing and are required for effective cutaneous repair^37^. Following adoptive transfer of Ly6C^hi^ monocytes, we found that 80.7±10.4% of donor-derived CD206+F4/80+ macrophages were CD301b+. Similarly, following adoptive transfer of Ly6C^lo^ monocytes, we found that 84.2± 6.4% of donor-derived CD206+F4/80+ macrophages were CD301b+. A greater frequency of donor-derived wound healing CD301b+CD206+F4/80+ macrophages was detected in animals receiving adoptively transferred Ly6C^lo^CD43^hi^ monocytes compared to those that received Ly6C^hi^CD43^lo^ monocytes (Fig. 4f). While F4/80 is present on all macrophages, co-expression of CD64 and MerTK exclusively distinguishes macrophages from monocytes^38^. These two populations significantly overlap, as 25.3 ± 2.1% of total F4/80+CD11b+ cells are also CD64+ MerTK+, but nearly all CD64+ MerTK+ cells are F4/80+ (Fig. 4c, Supplemental Figure S4c) and 88.9±1.2% of CD206+F4/80+ cells are also CD64+ MerTK+. Similarly, we detected a greater frequency of donor-derived CD301b+CD206+CD64+ MerTK+ macrophages in animals receiving adoptively transferred Ly6C^lo^CD43^hi^ monocytes, supporting the conversion of these monocytes into pro-regenerative macrophages (Supplemental Figure S4f). No changes in the frequency of CD301b−CD206+F4/80+ (Fig. 4f), CD301b−CD206+CD64+ MerTK+, or total number of macrophages (Supplemental Figure S4d,e,g) were detected between the two grafts. These results further support the hypothesis that circulating non-classical monocytes differentiate into wound repair macrophages.

### On-site delivery of FTY720 promotes accumulation of alternatively activated macrophages

**Figure 5:**
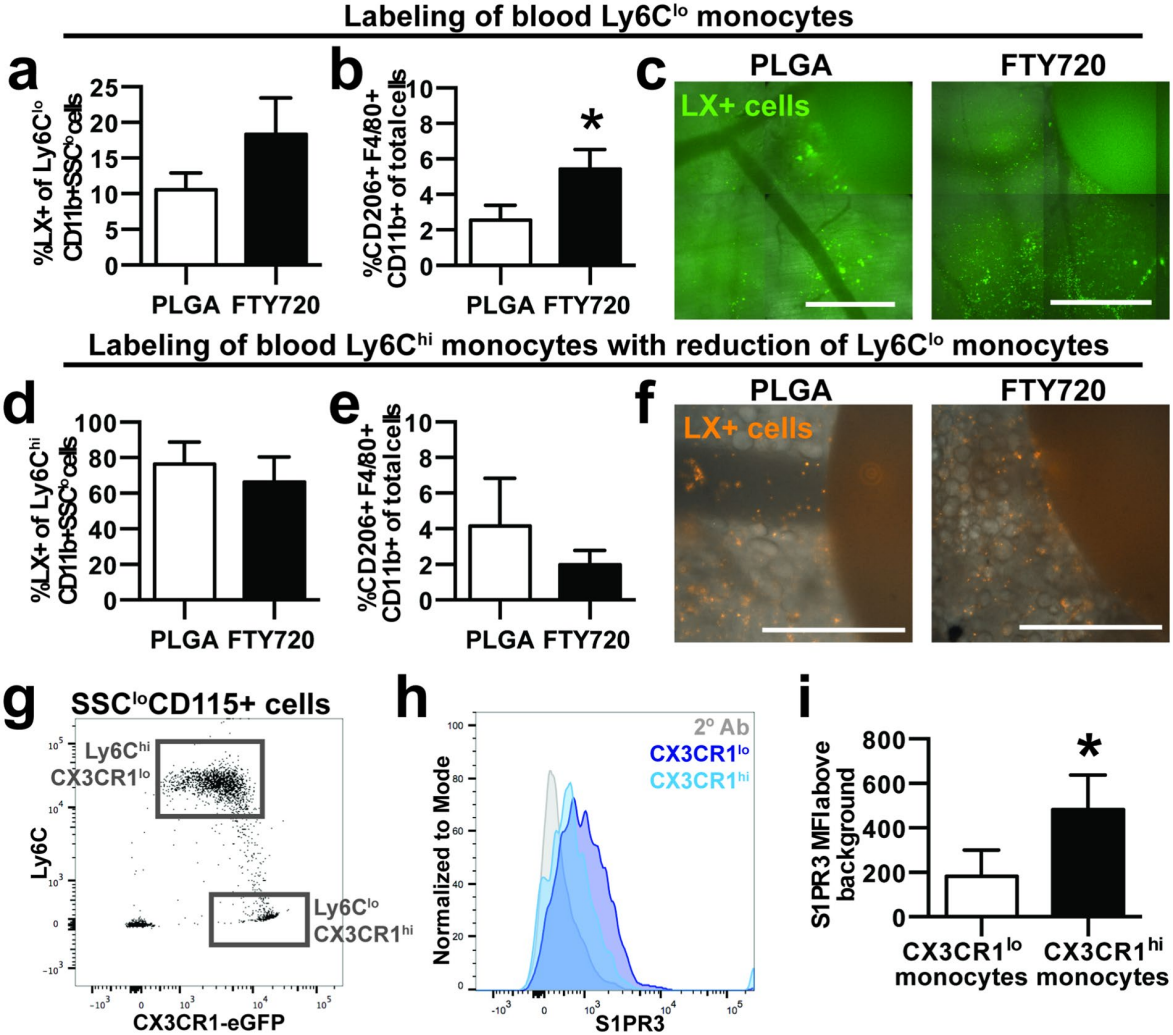
On-site delivery of FTY720 recruits blood-derived non-classical monocytes and increases the frequency of alternatively activated macrophages. PLGA films loaded with FTY720 were implanted immediately after DWC surgery. (**a**) Proportion of latex bead-positive (LX+) Ly6C^lo^ monocytes that were derived from blood in tissue 3 days post-injury following labeling of Ly6C^lo^ monocytes. (**b**) Overall frequency of CD206+ F4/80+CD11b+ macrophages in the presence of FTY720. (**c**) LX+ cells in tissue surrounding unloaded or FTY720-loaded PLGA implants. (**d**) Proportion of LX+ Ly6C^hi^ monocytes that were derived from blood following labeling of Ly6C^hi^ monocytes with Clod Lip. (**e**) Overall frequency of CD206+ F4/80+CD11b+ macrophages in the presence of FTY720 after Clod Lip administration. (**f**) LX+ cells in tissue surrounding unloaded or FTY720-loaded PLGA implants. (**g**) Ly6C expression in CX3CR1^hi^ monocytes and CX3CR1^lo^ monocytes obtained from the blood of CX3CR1^GFP/+^ mice identify Ly6C^lo^ monocytes and Ly6C^hi^ monocytes, respectively. (**h,i**) Surface S1PR3 expression in blood CX3CR1^hi^ cells and CX3CR1^lo^ cells. To control for background staining, CX3CR1^GFP/+^ cells were stained with secondary antibody (2° Ab) only, which is shown in the gray histogram. Scale bars, 500 µm. Data presented as mean ± S.E.M. Statistical analyses were performed using two-tailed t-tests. *p < 0.05, n = 4–10 animals per group.

We have previously demonstrated that localized delivery of the small molecule FTY720 from PLGA thin films enhances the recruitment of Ly6C^lo^ monocytes to inflamed peri-implant tissue and supports arteriogenesis^20, 34^. We explored the fate of specific blood monocyte populations in response to localized immune modulation by selectively labeling Ly6C^lo^ and Ly6C^hi^ monocytes followed by implantation of FTY720-loaded PLGA films in DWCs. Though statistically insignificant, a trend of increased frequency of blood-derived Ly6C^lo^ monocytes was observed with on-site delivery of FTY720 compared to blank implant (Fig. 5a,c; Supplemental Figure S5a). FTY720 increased the frequency of CD206+ macrophages within injured tissue 3 days post-surgery (Fig. 5b). The conversion efficiency of labeled blood-derived Ly6C^lo^ monocytes into CD206+ macrophages was similar between groups, suggesting that FTY720 does not enhance the rate at which monocytes convert to CD206+ macrophages (Supplemental Figure S5b). Selective labeling of Ly6C^hi^ monocytes demonstrates that FTY720 does not increase the frequency of circulation-derived Ly6C^hi^ monocytes in tissue when Ly6C^lo^ blood monocytes are reduced with clodronate liposomes (Fig. 5d,f). We observed no changes in the frequency of total CD206+F4/80+CD11b+ cells (Fig. 5e), suggesting that FTY720 is unable to increase the number of alternatively activated macrophages after reduction of circulating Ly6C^lo^ monocytes.

To investigate the molecular mechanisms of FTY720-mediated recruitment, we probed non-classical monocytes for expression of the sphingosine-1-phosphate receptor 3 (S1PR3), at which FTY720 exhibits agonist activity. We have previously demonstrated that FTY720 requires S1PR3 expression on hematopoietic cells in order to promote arteriogenic remodeling^20^. In addition to Ly6C, CX3CR1 (the fractalkine receptor) can be used to distinguish classical and non-classical blood monocytes^39^. While CX3CR1 is difficult to detect using antibody-based methods, CX3CR1^GFP/+^ transgenic mice enable real-time assessment of monocyte subset identity. CX3CR1^hi^ monocytes primarily overlap with the Ly6C^lo^ monocyte population, and conversely, CX3CR1^lo^ monocytes are primarily Ly6C^hi^ (Fig. 5g). S1PR3 surface expression is selectively higher on CX3CR1^hi^ blood monocytes (Fig. 5h,i; Supplemental Figure S5c), which indicates that elevated S1PR3 expression is a signature of non-classical monocytes and is consistent with our previous studies that demonstrated higher S1PR3 mRNA and total protein in Ly6C^lo^ monocytes^20^. Taken together, FTY720 likely increases the tissue content of CD206+ macrophages by recruiting circulating S1PR3^hi^ non-classical monocytes from circulation.

### CX3CR1^hi^ monocytes localize to a perivascular niche in wounded skin

**Figure 6:**
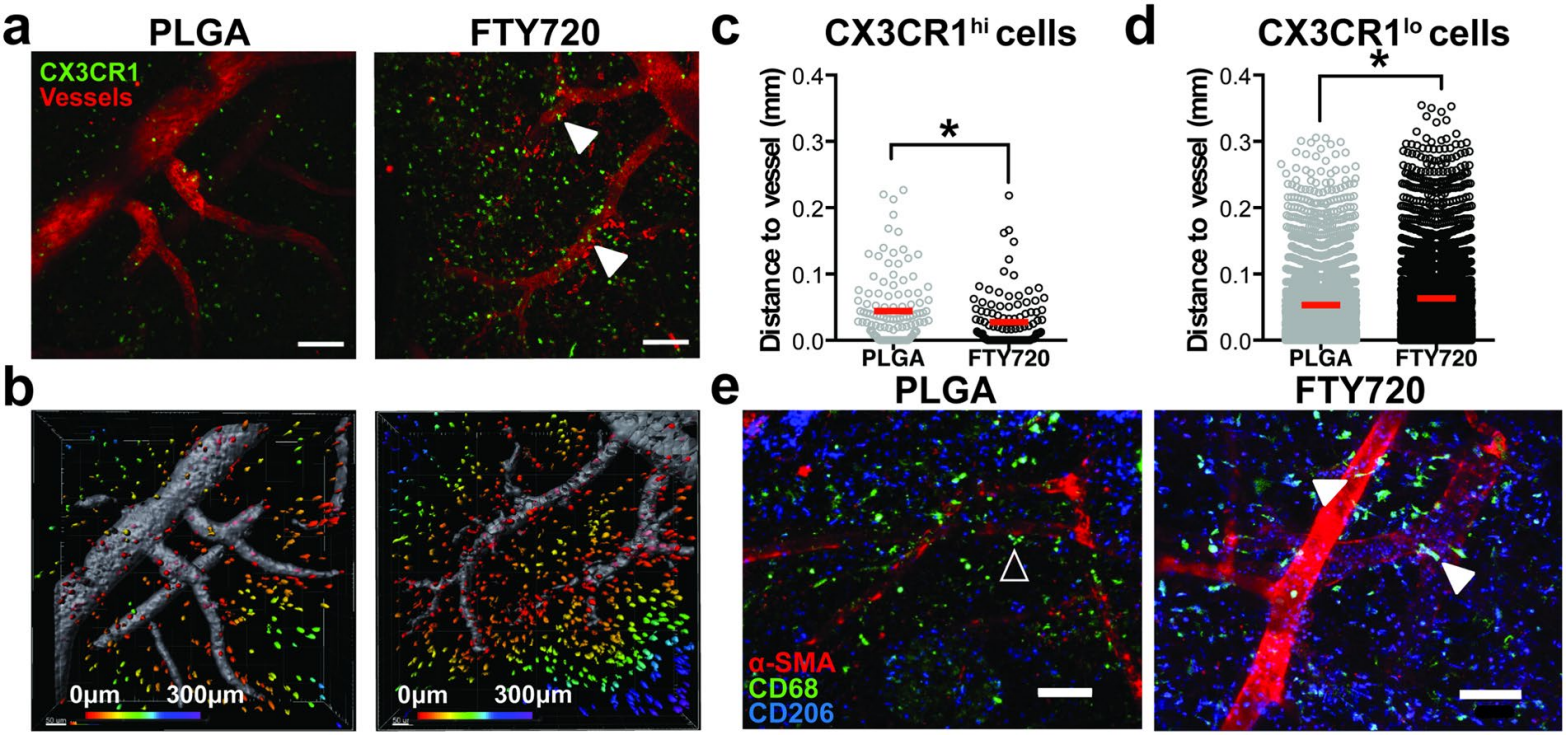
FTY720 recruits non-classical monocytes and CD206+ macrophages to perivascular niches in inflamed tissue. (**a**) Intravital imaging of DWCs in CX3CR1^GFP/+^ transgenic mice 1 day post-injury. CX3CR1^hi^ cells preferentially accumulated around peri-implant arterioles (white arrows) (**b**) Representative surface renderings of CX3CR1+ cells, color-coded according to distance from the closest blood vessel with red cells being closest to blood vessels and purple cells being the farthest away. Vessels were labeled with i.v. injection of rhodamine-dextran. (**c**) Distance to the nearest blood vessels of CX3CR1^hi^ cells in animals treated with FTY720 compared to unloaded PLGA controls. (**d**) Distance to the nearest blood vessels of CX3CR1^lo^ cells. Statistical analyses were conducted using two-tailed Mann-Whitney test. *p < 0.05, n > 100 cells, across 3–4 animals per group. (**e**) Immunostaining of CD68+CD206+ macrophages and vasculature. In animals treated with unloaded PLGA, perivascular CD68+CD206+ macrophages were circular (open arrow), while in FTY720-treated animals, CD68+CD206+ macrophages associated closely with inflamed vasculature and adopted an elongated morphology (solid white arrows, identified by aspect ratio greater than 2). Scale bars, 100 µm.

Non-classical monocytes are arteriogenic and can promote re-vascularization of damaged tissue^14, 20, 40^. To investigate the spatial distribution and function of monocytes in inflamed tissue, we performed intravital confocal microscopy of tissue surrounding FTY720-loaded implants 1 day post-surgery in CX3CR1^GFP/+^ mice. CX3CR1^hi^ and CX3CR1^lo^ cells were identified based on fluorescent intensity (Supplemental Figure S5d). Delivery of FTY720 from implanted films increased the frequency of non-classical CX3CR1^hi^ cells 1 day post-surgery (Fig. 6a) and significantly decreased the distance of CX3CR1^hi^ cells, but not CX3CR1^lo^ cells, to the nearest blood vessel (Fig. 6b–d). Additionally, CD68+CD206+ macrophages visualized by immunofluorescence adopted an elongated morphology (as determined by possessing an aspect ratio larger than 2) along the vasculature surrounding FTY720 implants (Fig. 6e). We have previously reported that FTY720 promotes expansion of peri-implant arterioles by 7 days post-injury^20, 41^. In the current studies, local delivery of FTY720 was able to induce modest expansion of arteriole microvessels (<50 µm diameter) by 3 days post-injury (Supplemental Figure S6a–c). Reduction of circulating non-classical monocytes with clodronate liposomes impaired arteriogenic expansion of peri-implant vessels (Supplemental Figure S6d). Interestingly, FTY720 exhibited a negative impact on vessel expansion when Ly6C^lo^ monocytes were reduced (Supplemental Figure S6d). Taken together, FTY720 recruits non-classical monocytes that convert into CD206+ macrophages that closely associate with peri-implant vasculature, where they support arteriogenesis.

**Figure 7:**
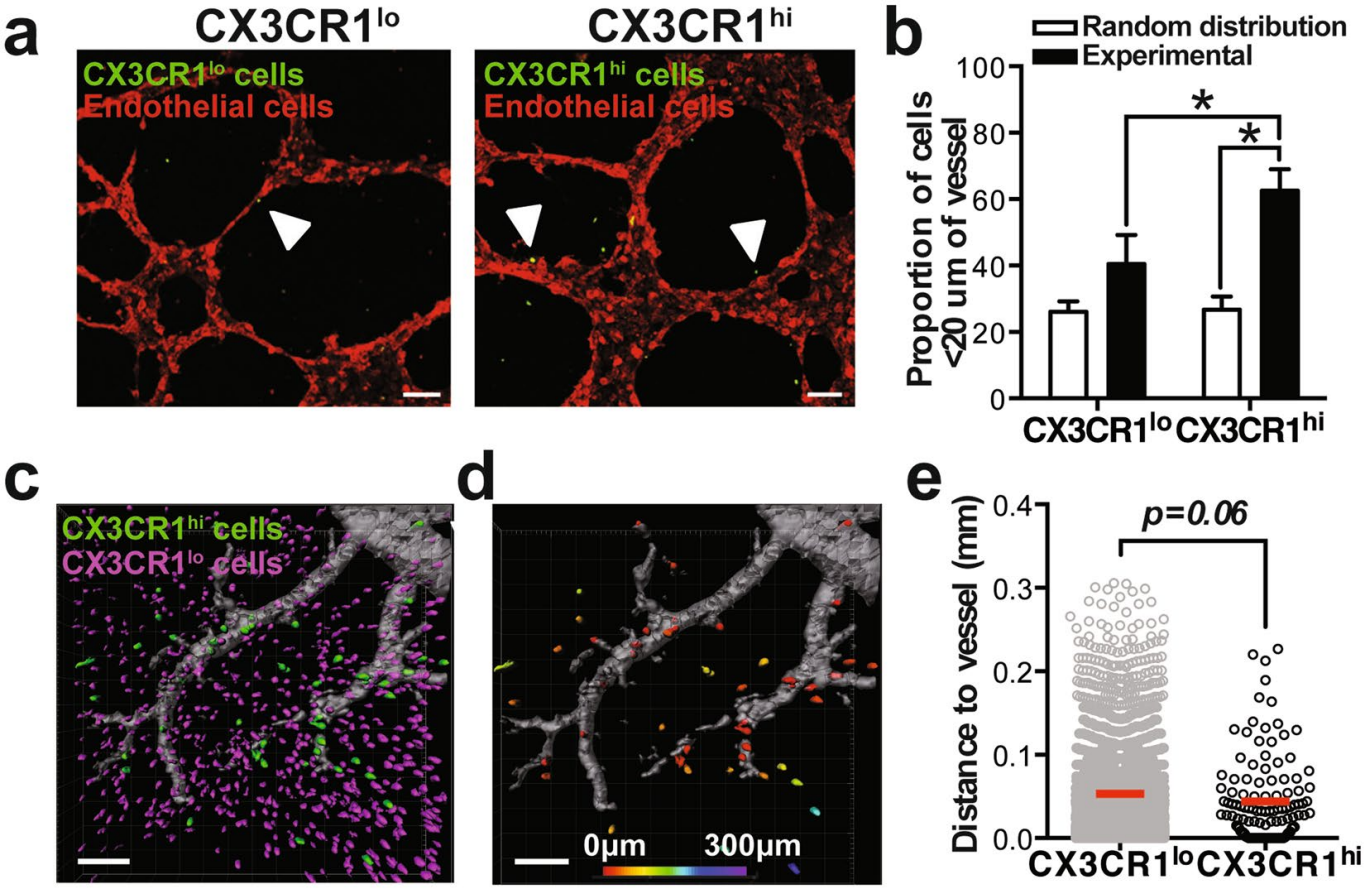
Non-classical monocytes localize to a perivascular niche. (**a**) Sorted CX3CR1^lo^ and CX3CR1^hi^ mouse monocytes associate with murine endothelial cell networks *in vitro* (solid white arrows) (**b**) The proportion of experimental CX3CR1^hi^ monocytes within 20 µm of the vessel network compared to CX3CR1^lo^ monocytes and a model-simulated random distribution of cells. Data presented as mean ± S.E.M. Statistical analyses were conducted using two-way ANOVA. *p < 0.05, n = 3 independent experiments. **(c)** Intravital imaging of CX3CR1^lo^ and CX3CR1^hi^ cells around PLGA implants in DWC mice 1 day post-surgery. **(d,e)** Distance of CX3CR1^lo^ and CX3CR1^hi^ cells to closest to blood vessel. Vessels were labeled with i.v. injection of rhodamine-dextran. Statistical analysis was performed using two-tailed Mann-Whitney test. *p < 0.05, n > 100 cells, across 3–4 animals per group. Scale bars, 100 µm.

To investigate whether perivascular localization is a signature of non-classical monocytes, we compared the spatial positioning of monocyte subsets *in vitro* and *in vivo*. CX3CR1^hi^ and CX3CR1^lo^ monocytes sorted from bone marrow of CX3CR1^GFP/+^ mice were co-cultured with murine endothelial cell networks on Matrigel. A significantly higher frequency of CX3CR1^hi^ non-classical monocytes compared to CX3CR1^lo^ classical monocytes localized to *in vitro*-forming vessel networks (Fig. 7a,b). To determine whether monocytes positioned themselves in proximity to the vessels, we compared the proportion of cells within 20 µm from the vessel to the positions of a computer-generated random distribution of cells. Random distributions were generated by stochastically positioning the same quantity of CX3CR1^hi^ or CX3CR1^lo^ monocytes on images of *in vitro* endothelial networks (Supplemental Figure S7). We were unable to distinguish randomly-generated distributions of cells from the experimental distribution of CX3CR1^lo^ monocytes with respect to the proportion of cells in close proximity (less than 20 µm) to the endothelial network (Fig. 7b). Conversely, the frequency of CX3CR1^hi^ monocytes in close proximity to the endothelial network was significantly higher than the random position distributions (Fig. 7b). Intravital confocal imaging surrounding FTY720-loaded polymer implants indicated that CX3CR1^hi^ cells were positioned closer to inflamed vasculature than CX3CR1^lo^ cells (Fig. 7c–e). These results indicate that non-classical monocytes preferentially localize near endothelial cells, which likely enables them to exert their angiogenic and arteriogenic effects^14, 25, 40^.

### FTY720 alters monocyte/macrophage accumulation and promotes vascular network expansion after arteriole ligation

**Figure 8:**
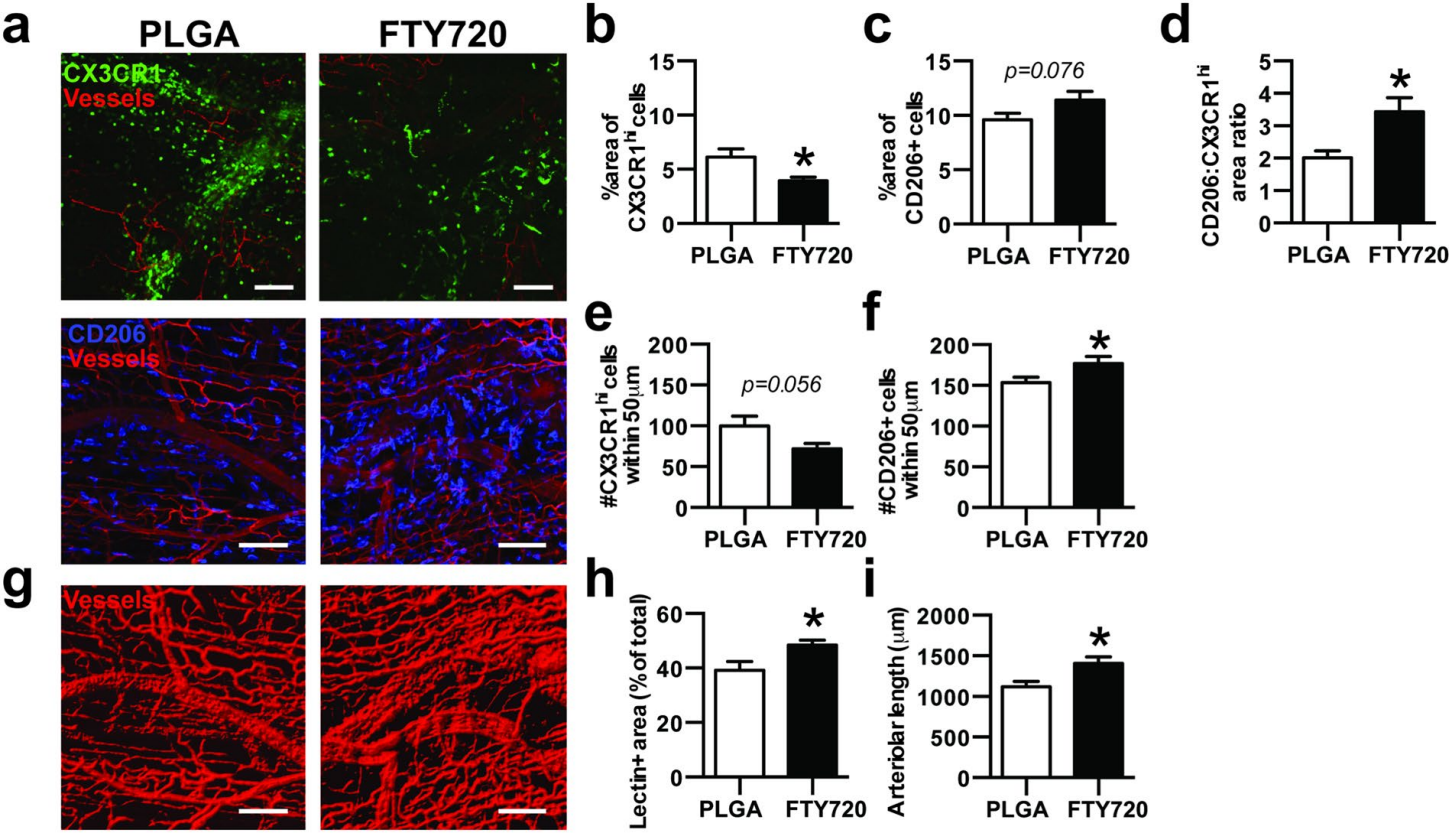
FTY720 increases CD206+ macrophages after arteriole ligation in the spinotrapezius muscle and promotes vascular network expansion. (**a**) Immunofluorescence of muscle tissue 3 days after ligation of spinotrapezius muscle arterioles in CX3CR1^GFP/+^ mice shows accumulation of CX3CR1^hi^ monocytes (top) and CD206+ macrophages (bottom) to lectin-stained vasculature 3 days post-ligation. (**b**) Area of CX3CR1^hi^ cells and (**c**) CD206+ cells in muscle tissue treated with unloaded PLGA or FTY720-loaded PLGA 3 days post-ligation. (**d**) Area ratio of CD206+ to CX3CR1^hi^ cells. (**e**) Number of CX3CR1^hi^ cells and (**f**) CD206+ cells within 50 μm of blood vessels. (**g,h**) Total density of lectin+ blood vessels and (**i**) the length of arterioles in the spinotrapezius muscle treated with FTY720 3 days post-ligation. Data presented as mean ± S.E.M. Statistical analyses performed using two-tailed t-tests. *p < 0.05, n = 25–27 FOVs from 5 animals per group. Scale bars, 100 µm.

The recruitment kinetics, fate, and function of myeloid cells during inflammation is heavily dependent on the specific type of tissue injury^18, 28^. Consequently, we sought to determine whether application of FTY720-loaded materials to ischemic muscle injury produces similar patterns of immunomodulation. Feeder arteriolar vessels within the murine spinotrapezius muscle were ligated in CX3CR1^GFP/+^ mice and unloaded or FTY720-loaded PLGA films were implanted over the muscle immediately after injury. We observed a decrease in the overall area of CX3CR1^hi^ cells in FTY720-treated animals 3 days post-injury (Fig. 8a,b). Conversely, we observed more CD206+ cells (Fig. 8c) and the area ratio of CD206+ cells to CX3CR1^hi^ cells was higher in FTY720-treated animals (Fig. 8d). Previous work has demonstrated that monocytes differentiate into alternatively activated macrophages in vascular niches^42^. We investigated the localization of CX3CR1^hi^ and CD206+ cells with respect to lectin-perfused vasculature. Fewer CX3CR1^hi^ cells (Fig. 8e), but more CD206+ cells (Fig. 8f) were found within 50 μm of blood vessels in FTY720-treated animals, which is consistent with perivascular conversion of non-classical monocytes into alternatively activated macrophages. Additionally, FTY720-treated animals had a greater vessel density and total length of arterioles 3 days after ligation (Fig. 8g–i).

## Discussion

**Figure 9:**
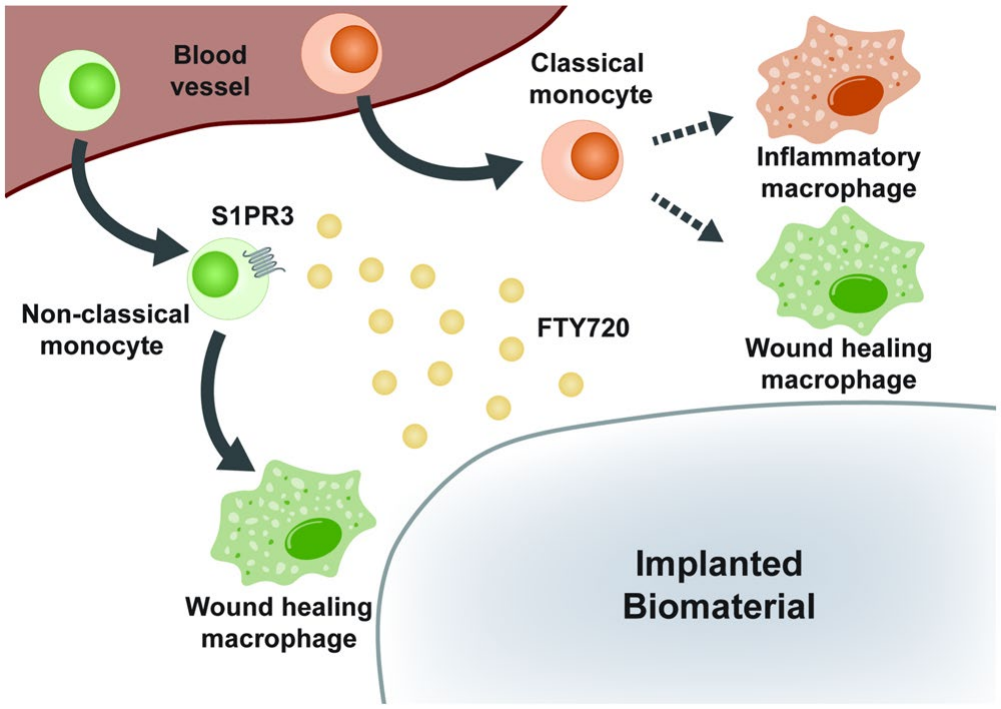
Local FTY720 release recruits biased progenitors of wound healing macrophages to inflamed tissue. Circulating non-classical S1PR3^hi^ monocytes are recruited by local delivery of FTY720 from a material implanted within an injury site. Upon entrance into inflamed tissue, non-classical monocytes give rise to alternatively activated, wound healing macrophages. Conversely, classical monocytes differentiate into both inflammatory and wound healing macrophages.

Monocytes are bloodborne mononuclear phagocytes that support tissue homeostasis and exit the vasculature at increased rates to differentiate into macrophages and dendritic cells during inflammation. The precise relationship of circulating classical and non-classical monocyte subsets to defined macrophage populations remains unknown. We have shown that after skin wounding and biomaterial implantation, circulating non-classical S1PR3^hi^ monocytes extravasate into inflamed tissue and serve as biased progenitors of CD206+CD301b+ wound healing macrophages. Previous work has demonstrated that classical monocytes directly convert to non-classical monocytes and macrophages within inflamed tissue^16, 19^. Therefore, these studies elucidate a complementary role for non-classical monocytes and argue that these cells primarily differentiate into alternatively activated macrophages. Biomaterial-mediated strategies that increase recruitment of non-classical monocytes through S1PR signaling are a promising strategy to increase accumulation of alternatively activated, wound healing macrophages (Fig. 9).

Non-classical Ly6C^lo^ monocytes patrol resting endothelium during homeostasis^12^, and are present in circulation at a slightly higher frequency than classical Ly6C^hi^ monocytes (Fig. 1b, Supplemental Figure S1b; 54.8 ± 7.5% Ly6C^lo^ monocytes vs. 30.4 ± 5.6% Ly6C^hi^ monocytes out of CD11b+SSC^lo^ cells). Interestingly, we observed a transient reduction of circulating monocyte populations 1 day post-surgery, which could be due to acute cell recruitment from the blood to injured tissue, as monocytopoiesis occurs over several days^43^. As previously reported^18^, we observed elevation of circulating myeloid cells 3 days post-injury, including total CD11b+ Ly6G-SSC^lo^ cells and Ly6C^hi^ and Ly6C^lo^ subpopulations (Fig. 1b). Though we detected a decrease in Ly6C^int^ monocytes 1 day post-injury, there was no elevation at 3 days post-injury, indicating that the size of this population may not be altered by inflammatory stimuli. The observed changes in circulating cell populations during inflammation are likely due in part to alterations in myeloid cell trafficking from the bone marrow, which displayed parallel changes in myeloid composition (Fig. 1c). We expect that these systemic changes in blood myeloid populations are a result of DWC surgery and not material implantation, as we have previously demonstrated that the number of rolling and adherent CX3CR1+ cells within dorsal skin vasculature is not significantly different between animals undergoing only DWC surgery and those that also received a PLGA implant^20^.

Intravascular non-classical Ly6C^lo^ monocytes orchestrate the disposal of necrotic endothelial cells after activation with TLR7-targeted danger signals during inflammation^12, 44^, whereas extravascular Ly6C^lo^ monocytes can promote angiogenesis and matrix remodeling via secretion of VEGF and matrix metalloproteinases^18^. The unique protein signature of non-classical monocytes, characterized by higher VEGF, TGFβ, and IL-10, and lower TNFα and IL-1β expression compared to classical monocytes has resulted in the suggestion that these cells may constitute a class of “anti-inflammatory” monocytes^18, 19^. Previous studies have indicated that Ly6C^lo^ monocytes in inflamed tissues such as skeletal muscle and focal hepatic injury are derived from cells recruited as Ly6C^hi^ monocytes from the blood and are converted *in situ* to Ly6C^lo^ monocytes to promote wound healing and tissue repair^16, 19^. Other reports suggest that Ly6C^lo^ monocytes are directly and robustly recruited from the blood, leading to extravascular accumulation during inflammation^12, 18, 31^. While we observed that blood-derived Ly6C^hi^ monocytes enter inflamed tissue, reduce Ly6C expression (Fig. 2g), and differentiate into macrophages (Figs 2i and 4f,g), our studies indicate that Ly6C^lo^ monocytes are also directly recruited from circulation to injured tissues and able to give rise to macrophages (Figs 2c and 4f). Previous studies have indicated that adoptively transferred Ly6C^lo^MHCII^−^ monocytes do not infiltrate the skin^13^, but this may be a result of differences in the grafted cell population (Ly6C^lo^MHCII^−^ monocytes vs. Ly6C^lo^CD43^hi^ monocytes in our studies) or inflammatory stimuli (LPS injection vs. excisional skin injury). Though we observed a decrease in Ly6C^lo^ blood monocytes after clodronate liposome administration, we did not detect a difference in the frequency of Ly6C^lo^ monocytes within injured skin. Many studies have shown that Ly6C^hi^ monocytes can convert to Ly6C^lo^ monocytes in the blood or tissue^11, 19, 29^. Similarly, our data may reflect that in the absence of Ly6C^lo^ monocytes, circulation-derived Ly6C^hi^ monocytes compensate and increase their rate of conversion into Ly6C^lo^ monocytes in inflamed tissue.

An important finding of our work is that circulating Ly6C^lo^ monocytes preferentially contribute to the CD206+ wound macrophage pool within skin injury compared to Ly6C^hi^ monocytes. Analysis of peri-implant tissue for recruited latex bead-labeled Ly6C^lo^ monocytes shows that this subset is predisposed to acquire a CD206+ alternatively activated M2-like^45^ macrophage phenotype (Figs 2e and 4f). Administration of clodronate liposomes enables selective labeling of Ly6C^hi^ monocytes, while simultaneously reducing the frequency of Ly6C^lo^ monocytes for 2–7 days after administration^11, 35^. Clodronate liposome-treated animals displayed no change in the frequency of overall macrophages within the skin injury after 3 days, but exhibited a decrease in the proportion of CD206+ macrophages (Fig. 3c,e). Because tissue monocyte composition was unaffected by clodronate liposomes, these results may reflect a delay in generation of CD206+ macrophages. We cannot exclude the possibility that transient monocyte depletion alters myeloid cell responses and recruitment during inflammation, as monocyte subsets are known to communicate with each other^5^.

Latex bead-based labeling strategies are useful because they overcome many of the limitations of adoptive transfer, including *ex vivo* cell manipulation, pooling of donor cells, and low sensitivity due to poor cell recovery^46^. Importantly, there is no evidence that these labeling techniques alter monocyte recruitment or systemic inflammation^46^. However, our interpretation of these studies relies on retention of latex beads within the cell that was originally labeled and no transfer to other cells. Therefore, cell tracking is best performed by use of complimentary tracking methods, including adoptive transfer, selective labeling techniques, and transgenic or knockout mice. We adoptively transferred 5.55 × 10^5^ CD45.1+ Ly6C^hi^ or Ly6C^lo^ monocytes by intravascular injection into CD45.2 mice at the time of DWC surgery. We did not detect differences in the total number of CD45.1+ donor-derived cells per milligram of dorsal tissue between the two monocytes grafts, and further probed for markers of macrophage differentiation and polarization within donor cells. CD301b+ CD206+ macrophages drive midstage skin regeneration by promoting fibroblast repopulation, cellular proliferation, and re-epithelialization^37^. In our adoptive transfer studies, a higher frequency of donor Ly6C^lo^ monocytes acquired a CD301b+ CD206+ macrophage phenotype compared to donor Ly6C^hi^ monocytes (Fig. 4f), indicating that circulation-derived Ly6C^lo^ monocytes may be intrinsically predisposed to convert to dermal wound healing macrophages. Conversely, there was no difference in the frequency of donor-derived CD301b−CD206+ or number of total macrophages between mice receiving Ly6C^hi^ or Ly6C^lo^ monocytes (Fig. 4g, Supplemental Figure S4). These data, along with the observation that FTY720 administration increases CD206+ macrophages within injured skin (Fig. 5b), further supports the hypothesis that non-classical monocytes undergo local conversion into alternatively activated macrophages.

While macrophages are key mediators of the host response to implanted materials that govern the integration of implants into host tissue^47, 48^, the role that their monocytic precursors play in regulating implant outcome has been largely unexplored. In our studies, around 45% of monocytes surrounding PLGA implants were Ly6C^lo/int^. In previous work^21^, we demonstrated that approximately 40% of monocytes around heparin-containing PEG hydrogels were Ly6C^lo/int^. While lymphocyte, granulocyte, and macrophage accumulation are impacted by the type of material implanted^49^, future studies are needed to determine how different classes of materials impact infiltration of monocyte and macrophage subsets. Interestingly, we observed model-specific differences in the kinetics of monocyte recruitment during FTY720 delivery. While FTY720 increases the frequency of Ly6C^lo^ monocytes 3 days after skin wounding^20^ (Fig. 5a), we observed a decrease in CX3CR1^hi^ monocytes 3 days after arteriole ligation in the spinotrapezius muscle (Fig. 8b). The progression of inflammation may be expedited during ischemia because of the need to rapidly restore blood supply and oxygen transport. FTY720 increases the frequency of F4/80+ CD206+ macrophages both in wounded skin (Fig. 5b) and CD68+CD206+ macrophages in ischemic muscle (Fig. 8f). Previously, we have detected more M2-like macrophages within mandibular bone defects 3 weeks after implantation of FTY720-loaded polymer nanofibers^50^. Taken together, these findings suggest that FTY720 delivery from different biomaterials can enhance pro-regenerative myeloid cell recruitment, but the kinetics may vary depending on the type of injury.

Monocytes and macrophages have been shown to interact closely with remodeling vasculature^51–53^. *In vitro*, CX3CR1^hi^ monocytes preferentially associated with endothelial cell networks (Fig. 7a,b), and *in vivo*, exhibit closer homing to inflamed vasculature (Fig. 7c–e). While further studies are needed, it is possible that the vasculature provides microenvironmental cues that educate non-classical monocytes to convert into alternatively activated macrophages via signaling with the endothelium^42^. Conversely, monocytes and macrophages appear to promote both angiogenic and arteriogenic expansion of the vasculature^14, 52, 54^. Macrophages are the primary source of angiogenic growth factors such as VEGF^55^ and robust vascularization is associated with increased macrophage presence in and around biomaterial implants^33, 50^. M2-polarized macrophages (IL-4- or IL-10-stimulated) are considered pro-angiogenic both *in vitro* and *in vivo* ^56–58^, though recent studies have demonstrated that M1-polarized macrophages may also play key roles in angiogenesis^59, 60^. Promotion of both angiogenic expansion of vascular length and arteriogenic diameter network expansion in dorsal skin is correlated with the presence of Ly6C^lo^ monocytes in higher proportion to Ly6C^hi^ monocytes^21^. Hydrogel vascularization in response to growth factor delivery (platelet-derived growth factor and fibroblast growth factor) in the cornea was accompanied by accumulation of macrophages that produce both M1- and M2-associated mRNA transcripts, such as *Tnfa* and *Arg1*, respectively^33^. We found that ischemic muscles treated with FTY720 have increased numbers of perivascular CD206+ cells located (Fig. 8f), which coincided with increased arteriolar length and total vessel density (Fig. 8g–i). FTY720 likely acts on both immune cells and the endothelium, as loss of S1PR3 in hematopoietic or parenchymal cells impairs FTY720-induced arteriogenic remodeling^20^. Delivery of FTY720 similarly induced both increased accumulation and perivascular localization of alternatively activated macrophages after volumetric muscle loss, which was accompanied by enhanced re-vascularization and muscle healing^61^. Consequently, both the phenotype of monocytes and macrophages, as well as their spatial distribution is likely an important feature when assessing their function.

Taken together, our studies shed light on the functions of non-classical monocytes during inflammation and wound healing. Monocytes and macrophages are increasingly appreciated for their roles in regulating tissue homeostasis and coordinating repair after damage. Acute modulation of the inflammatory response has been shown to regulate repair of tissue at longer time scales, including bone^50^ and skeletal muscle^19^. An understanding of the origin of monocyte and macrophage populations during wound healing and the cues governing their *in situ* fate is critical to harnessing endogenous mechanisms of repair. This work provides new insights into the origin of alternatively activated wound healing macrophages that can be leveraged to develop next-generation immunoregenerative biomaterials capable of finely tuning the inflammatory response.

## Materials and Methods

### Material fabrication

Films were fabricated as previously described^20^. Briefly, 350 mg PLGA (50:50 DLG 5E – Evonik Industries) was dissolved in 2ml dichloromethane in a glass scintillation vial via high-speed vortexing. For drug-loaded films, 1.75 mg of FTY720 (Cayman Chemical) was added at a 1:200 drug:polymer weight ratio, and mixed until completely incorporated. Polymer solutions were poured into Teflon-coated petri dishes and allowed to dry at −20 °C for 7 days. Before use, films were lyophilized overnight to remove any traces of solvent.

### Dorsal skinfold window chamber surgery

All animal procedures were conducted according to protocols approved by the Georgia Institute of Technology or University of Virginia Institutional Animal Care and Use Committee. Male C57BL/6J or B6.129P-Cx3cr1tm1Litt/J mice (CX3CR1^GFP/+^) mice (8–12 weeks) were fitted with sterile dorsal skinfold window chambers (APJ Trading Co) as previously described^20^. Briefly, mice were anesthetized with an intraperitoneal (i.p.) injection of ketamine/xylanzine (100/10 mg/kg) in sterile saline. Dorsal skin was shaved, depilated, and sterilized with three alternating washes of 70% ethanol and chlorhexidine. A double-layered skin fold was elevated off the back of the mouse and fitted with the titanium frame of the window chamber on the underside. The epidermis and dermis were removed from the top side of the skinfold in a ~12 mm diameter circular area via surgical microscissors to reveal underlying vasculature. Exposed tissue was superfused with sterile saline to prevent desiccation. The titanium frame was then mounted on the top side of the skinfold, attached to the underlying frame counterpart, and sutured to the surrounding tissue. Two films were placed on top of the exposed subreticular dermis layer immediately after surgery (day 0) and exposed tissue was sealed with a sterile glass window. Mice were euthanized 72 hours after surgery via CO_2_ asphyxiation. The vasculature was immediately flushed with intracardiac infusion of saline followed by an intracardiac infusion of 4% paraformaldehdyde, prior to whole mounting of tissue and immunohistochemistry.

### Spinotrapezius ischemia model

Mice were anesthetized with an i.p. injection of ketamine⁄xylazine⁄atropine (60⁄4⁄0.2 mg⁄kg). Ligation surgeries were performed as previously described^51^. Briefly, a small incision was made on the dorsum above the lateral edge of the right spinotrapezius at the edge of the fat pad. The fascia was separated from the top of the muscle and the fat pad moved before isolating an anatomically reproducible feeding arteriole entering the muscle from below. This feeding arteriole was ligated with 10-0 nonabsorbable suture in two places and cut. The fat pad and fascia were moved back into position and the skin was closed with 8-0 nonabsorbable suture. To allow visualization of vascular endothelium of arterioles in CX3CR1^GFP/+^ mice, anesthetized mice were administered an intra-jugular injection of labeled isolectin (IB4-Alexa Fluor 568; Life Technologies), which was allowed to circulate for 10 minutes. Anesthetized mice were euthanized via CO_2_ asphyxiation 72 hours post-surgery. The vasculature was immediately flushed with an intracardiac infusion of adenosine (70 mg/L) in Ringer’s solution followed by an intracardiac infusion of 4% paraformaldehyde.

### Flow cytometry

Peripheral blood was collected via cardiac puncture and bone marrow was collected via centrifugation (1000 g for 5 mins) of isolated tibiae. The dorsal tissue circumscribing films was punched out with 6 mm biopsy punches and pooled from both films within one animal for most studies. For adoptive transfer studies, all inflamed dorsal tissue was collected for analysis. Tissue was digested with collagenase (1 mg/ml) at 37 °C for 30 minutes and further disaggregated with a cell strainer to create a single cell suspension. Single cell suspensions of tissues were stained for flow cytometry analysis according to standard procedures and analyzed on a FACS-AriaIIIu flow cytometer (BD Biosciences). The following antibodies were used for cell phenotyping: APC-Cy7- or BV421-conjugated anti-CD11b (BioLegend), APC- or BV510-conjugated anti-Ly6C (BioLegend), PerCP-Cy5.5-conjugated anti-CD43 (Biolegend), PE-Cy7-conjugated anti-GR-1 (BioLegend), APC-Cy7-conjugated anti-Ly6G (Biolegend), APC-conjugated anti-F4/80 (BioLegend), PE-Cy7- or FITC-conjugated anti-CD206 (BioLegend), PE-Cy7-conjugated anti-CD301b, BV711-conjugated anti-CD64 (BioLegend), PE-conjugated anti-MerTK (Biolegend), BV605-conjugated anti-CD45.1 (Biolegend), BV785-conjugated anti-CD45.2 (Biolegend), or PerCP-eFluor710 conjugated anti-CD115 (eBioscience). Dead cells were excluded by staining with Zombie NIR^TM^ (Biolegend) in protein-free buffer prior to antibody staining. Staining using BV dyes was performed in the presence of Brilliant Stain Buffer (BD Biosciences). Positivity was determined by gating on fluorescence minus one controls. Absolute quantification of cell numbers in blood and tissue was performed by adding 25 μL of AccuCheck counting beads to flow cytometry samples (Thermo Fisher Scientific). S1PR3 flow cytometry was performed by first performing Fc block (Biolegend), followed by staining cells with primary unconjugated anti-S1PR3 antibody (Alomone Labs) and secondary staining with DyLight 650 anti-rabbit IgG (Abcam). Positivity was determined by staining CX3CR1^GFP/+^ cells with the secondary body only (no primary S1PR3 antibody).

### Cell tracking of Ly6C^lo^ and Ly6C^hi^ monocytes

For selective labeling of Ly6C^lo^ monocytes, mice were administered 250 µL of Fluoresbrite® Polychromatic Red latex beads intravenously one day prior to surgery (0.5 μm, Polysciences - diluted 1:25 in sterile saline) via jugular vein injection. For selective labeling of Ly6C^hi^ monocytes, mice were administered 100 μL of clodronate liposomes per 10 g of mouse body weight (Dr. Nico van Rooijen, clodronateliposomes.com) intravenously two days prior to surgery, followed by administration of latex beads 16 hours later (one day prior to surgery). Labeling was confirmed by retro-orbital blood draw days 1 and 3 post-surgery. For adoptive transfer studies, white blood cells from bone marrow, spleen, and blood were collected from mice expressing the CD45.1 allelic variant and enriched for monocytes using an EasySep Mouse Monocyte Isolation Kit (Stem Cell Technologies). Cells were further purified by fluorescence activated cell sorting on a BD FACS AriaII cell sorter using the following markers: Ly6C^hi^ monocytes (SSC^lo^CD11b^+^Ly6C^hi^CD43^lo^) or Ly6C^lo^ monocytes (SSC^lo^CD11b^+^Ly6C^lo^CD43^hi^). Mice received 555,000 Ly6C^hi^ or Ly6C^lo^ monocytes via jugular vein injection on the day of surgery.

### Intravital image acquisition

Mice were anesthetized with isofluorane, the glass window was removed, and dorsal tissue was superfused with saline to prevent desiccation. Up to two films were implanted into the window chambers on the day of surgery (day 0). To label perfused vasculature, mice were anesthetized with isoflurane and given a retro-orbital injection of high molecular weight TRITC-conjugated dextran (2 MDa; Life Technologies). For imaging, the anesthetized mouse was secured to the microscope stage in a custom adapter, the glass window was removed, and dorsal tissue was superfused with sterile saline. Intravital confocal microscopy was conducted with a 20X water immersion objective (NA = 1.0) on a Zeiss LSM710 NLO microscope and z-stack images were acquired immediately proximal to the films. For 3D analysis in Imaris (Bitplane), images of 708 × 708 µm regions were acquired adjacent to the implant to visualize immune cell distribution in the close surrounding tissue. Cells expressing CX3CR1-GFP were identified in Imaris using the surface tool. CX3CR1+ surfaces were identified by smoothing with a 2 μm grain size and an automatic threshold on absolute intensity. Touching objects were split using a seed points diameter of 10 μm. CX3CR1^hi^ versus CX3CR1^lo^ cells were discriminated by applying a filter to select surfaces with a high fluorescence intensity in the CX3CR1-GFP channel (above 150 max intensity). Vessels were identified in Imaris by drawing a surface on the TRITC-dextran fluorescent channel with a 3 μm grain size, manually-selected threshold value (determined based on each image), and manually-selected volume filter to remove small debris. To calculate the distance between CX3CR1+ cells and the nearest blood vessel, a distance transformation was applied to TRITC-dextran vessel surfaces and the median position of each CX3CR1+ cell within this space was recorded.

### Whole mount immunohistochemistry

Dorsal tissue and spinotrapezius muscles were explanted and permeabilized overnight with 0.1–02% saponin. The tissues were blocked overnight in 5–10% mouse serum. Tissues were incubated at 4 °C overnight in solution containing 0.1% saponin, 5% mouse serum, 0.5% bovine serum albumin, and the following conjugated fluorescent antibodies: Alexa Fluor 594 anti-CD31 antibody (BioLegend) or Alexa Fluor 568 isolectin IB4 (Life Technologies) for blood vessel visualization, Alexa Fluor 647 anti-CD68 (AbD Serotec) for monocyte/macrophage visualization, and Alexa Fluor 488 anti-CD206 (AbD Serotec) or Alexa Fluor 647 anti-CD206 (Biolegend). Tissues were mounted in 50/50 glycerol/phosphate buffered saline and imaged through the entire thickness of the muscle (~200 microns) on a Zeiss LSM 710 NLO confocal microscope or a Nikon confocal microscope.

For monocyte/macrophage quantification, 3–4 different fields of view (FOVs) per muscle containing a collateral arteriole with monocytes/macrophages evident were located manually. Full-thickness z-stack (2 μm step size) volume renders of these FOVs were generated using a 20X oil immersion objective. 20X confocal z-stack volume renders were used for cell association quantification. A threshold at which only the brightest CX3CR1^GFP/+^ cells (CX3CR1^hi^) were visible was applied to all images. Vessel-associated CD206+ and CX3CR1^hi^ cells were defined as cells falling within the 2-dimensional area of the vessel in question and within 50 μm of the vessel border. ImageJ (NIH) imaging software was used to quantify vessel length and density. Arterioles were distinguished from venules by degree of lectin binding, vessel size, and morphology. Cell counts and green channel thresholding to designate CX3CR1^hi^ cells were performed in Adobe Photoshop (Adobe Systems Incorporated).

### Angiogenesis assay with monocyte co-culture

C166 murine yolk-sac endothelial cells (ATCC) were propagated in flasks coated with 0.1% gelatin (Stem Cell Technologies) using endothelial growth medium (Angioproteomie) and incubated at 37 °C and 5% CO_2_ atmosphere. Monocytes were isolated from bone marrow of male CX3CR1^GFP/+^ mice and sorted using a FACS-Aria IIIu to discriminate CX3CR1^hi^ monocytes and CX3CR1^lo^ monocytes. Endothelial tube forming assays were performed in 15-well angiogenesis slides (Ibidi) as follows. Wells were coated with BD Matrigel^TM^ and seeded with C166 cells (7500 cells/well) and CX3CR1^hi^ or CX3CR1^lo^ monocytes (2000 cells/well). After 20 hours, co-cultures were fixed and labeled with rhodamine-phalloidin. Images were captured using a Zeiss LSM 700 confocal (10x magnification) and Zen software (Zeiss). The centroid of each monocyte was identified and the distance to the nearest endothelial tube formation was calculated using a custom MATLAB code. For each vessel image, a random distribution of “cells” was overlaid on the image (equal to the number of monocytes in the original image) and the distance of the “random cells” to the nearest vessel was calculated. The experimental monocyte distance to vessel was then compared to the computer-generated randomized cell distance to vessel to determine whether the cells preferentially distribute themselves near vessel segments.

### Statistical analysis

Data are presented as mean ± standard error of the mean (S.E.M.). All statistical analysis was performed in GraphPad Prism software. Comparisons of two groups were made using a two-tailed unpaired t-test, with Welch’s correction if standard deviations were not equal. For studies with two independent variables, two-way ANOVA with Sidak’s test for multiple comparisons was performed. For confocal intravital microscopy (Fig. 6), data reflect eight ROIs acquired across 3–4 animals per group, and statistical comparisons were made using a two-tailed Mann-Whitney test. Unless otherwise noted, *p* < *0.05* was considered statistically significant.

## Electronic supplementary material

Supplementary Information
