# Supplementary material for: Non-classical monocytes are biased progenitors of wound healing macrophages during soft tissue injury
Source: Sci Rep. 2017 Mar 27;7:447. doi: 10.1038/s41598-017-00477-1 (PMC5428475; doi:10.1038/s41598-017-00477-1)
Supplement: Supplementary file 1 — Supplementary Information [file 41598_2017_477_MOESM1_ESM.pdf]

Title: Non-classical monocytes are biased progenitors of wound healing macrophages during soft tissue injury

Authors: Claire E. Olingy[a,1], Cheryl L. San Emeterio[a,1], Molly E. Ogle[a], Jack R. Krieger[a], Anthony C. Bruce[b], David D. Pfau[a], Brett T. Jordan[a], Shayn M. Peirce[b], and Edward A. Botchwey[a,*]

Affiliations:

[a]Wallace H. Coulter Department of Biomedical Engineering; Georgia Institute of Technology and Emory University; 315 Ferst Drive, Atlanta, GA 30332, USA

[b]Department of Biomedical Engineering; University of Virginia; Box 800759, Charlottesville, VA 22908, USA

***Corresponding Author Contact Information:**

Edward Botchwey

Department of Biomedical Engineering, Georgia Institute of Technology

315 Ferst Drive

Atlanta, Ga 30332

Email: edward.botchwey@bme.gatech.edu

Phone: (404) 385-5058

Additional Footnotes:

[1]These authors contributed equally to this work.

Supplemental Information

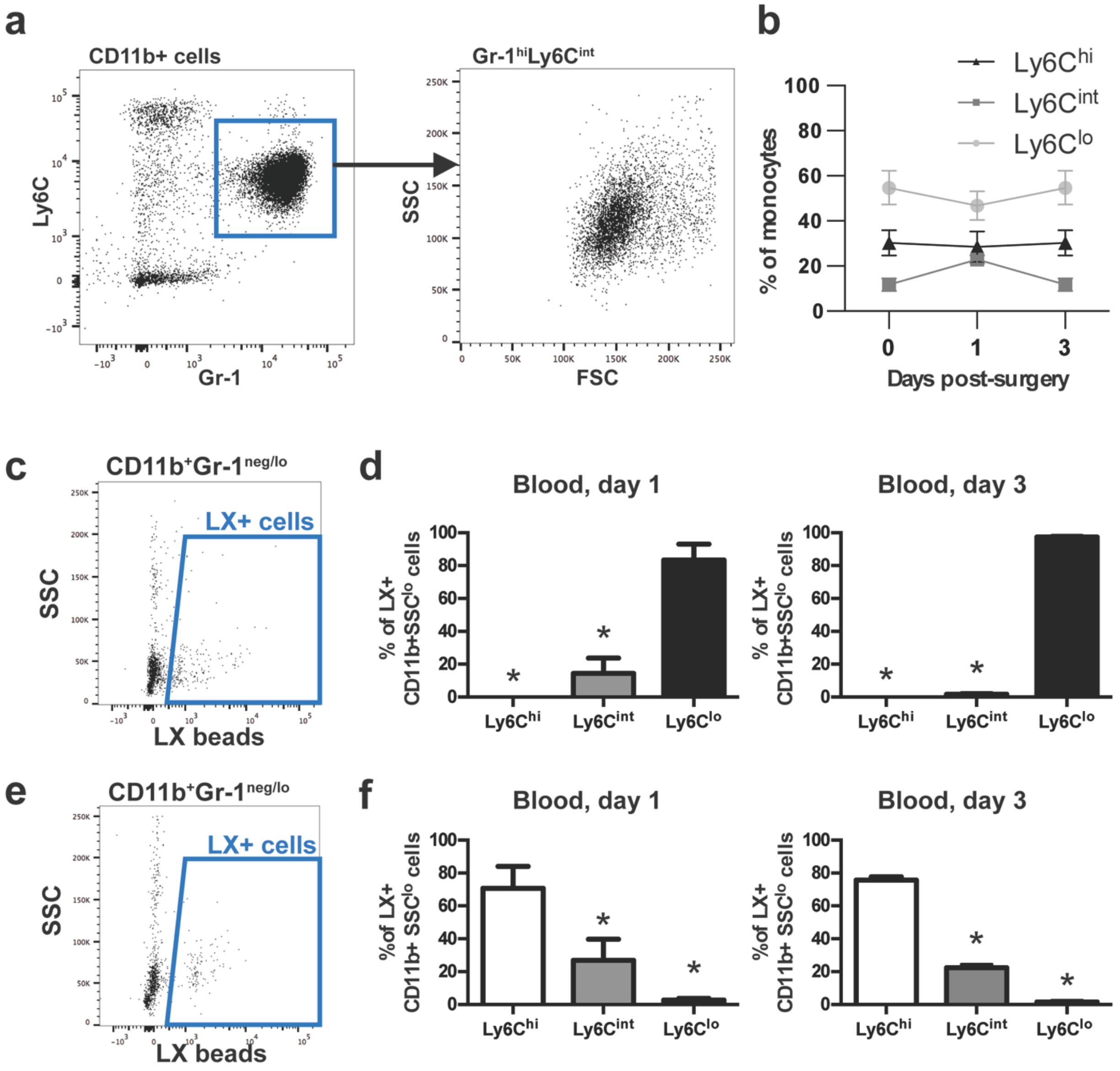

Supplemental Figure S1. **(a)** Ly6C^{int}Gr-1^{int} cells are SSC^{hi} granulocytes/neutrophils that were removed from subsequent monocyte gating. **(b)** Ly6C^{lo} monocytes were more abundant than Ly6C^{int} and Ly6C^{hi} monocytes during the first 3 days after dorsal skinfold window (DWC) surgery. The overall frequency of monocyte subsets within the monocyte gate (CD11b+Gr-1^{lo}SSC^{lo}) did not change over time. **(c-d)** Latex beads were injected into the blood 24 hours prior to DWC surgery. Analysis of blood at days 1 and 3 post-injury shows selective labeling of Ly6C^{lo} monocytes. **(e-f)** Clodronate liposomes were administered 2 days before DWC surgery and Ly6C^{hi} monocytes that rapidly return to circulation were labeled with latex beads 16 hours post-depletion. Analysis of blood at days 1 and 3 post-injury shows selective labeling of Ly6C^{hi} monocytes. Data presented as mean ± S.E.M. Statistical analyses were conducted using two-tailed t-tests. *$p<0.05$ compared to Ly6C^{lo} (d) or Ly6C^{hi} monocytes (f), n=4-11 animals per group.

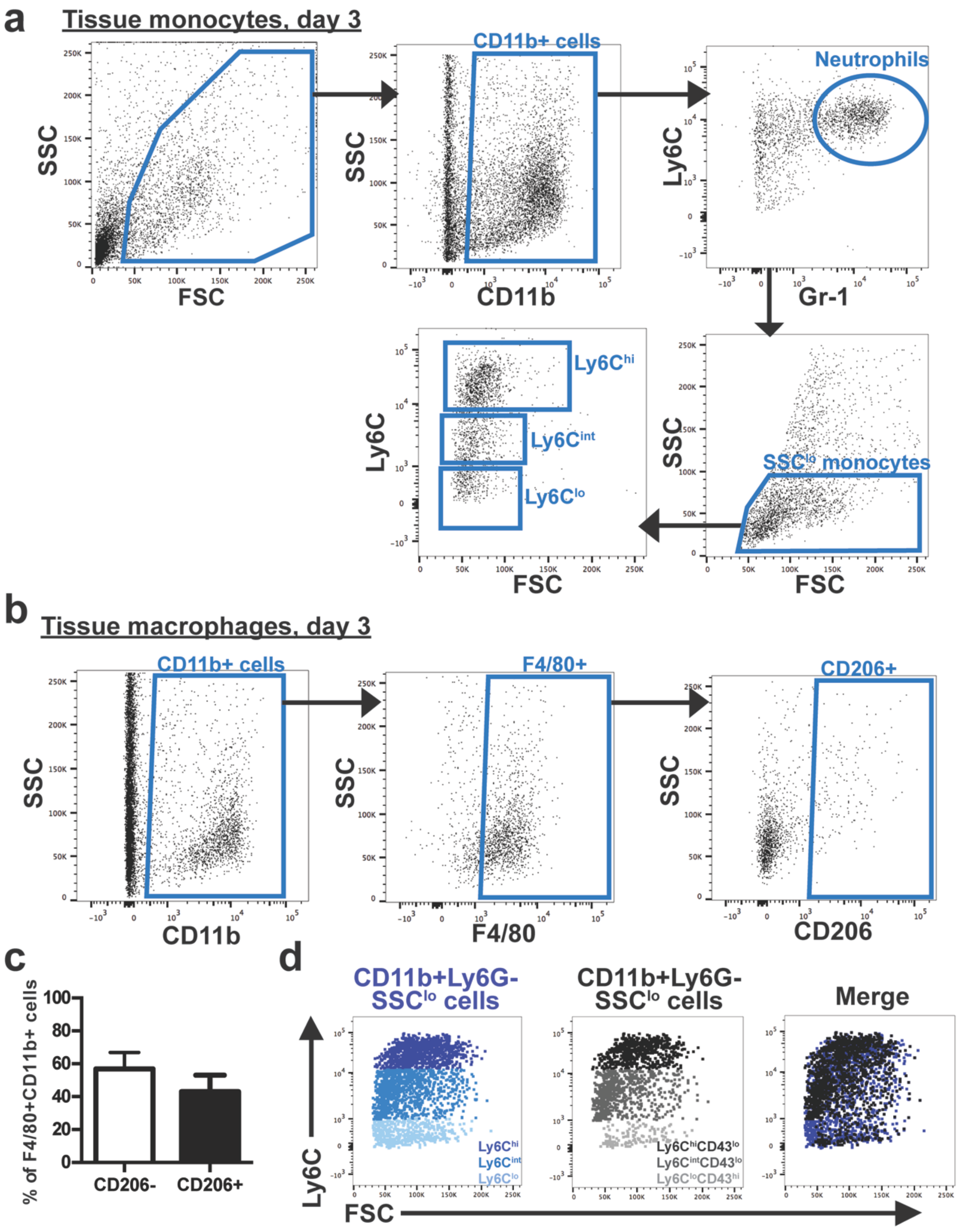

Supplemental Figure S2. **(a)** Gating strategy for tissue monocytes at 3 days post-injury. Tissue cells immunophenotyped as CD11b+Ly6C^{int}Gr-1^{hi} were excluded to remove granulocytes. Remaining CD11b+ cells were gated on SSC^{lo} and Ly6C expression for monocyte subpopulation analysis. **(b)** Gating strategy for tissue macrophages at 3 days post-injury. Tissue cells were gated on CD11b+F4/80+ cells to identify macrophages and subsequently assessed for CD206 expression for macrophage subpopulation analysis. **(c)** Total CD11b+F4/80+ cells collected for dorsal tissue 3 days post-injury are about half CD206+. **(d)** CD43 further discriminates CD11b+Ly6G-SSC^{lo} monocyte subpopulations, but primarily overlaps with the populations identified using only Ly6C.

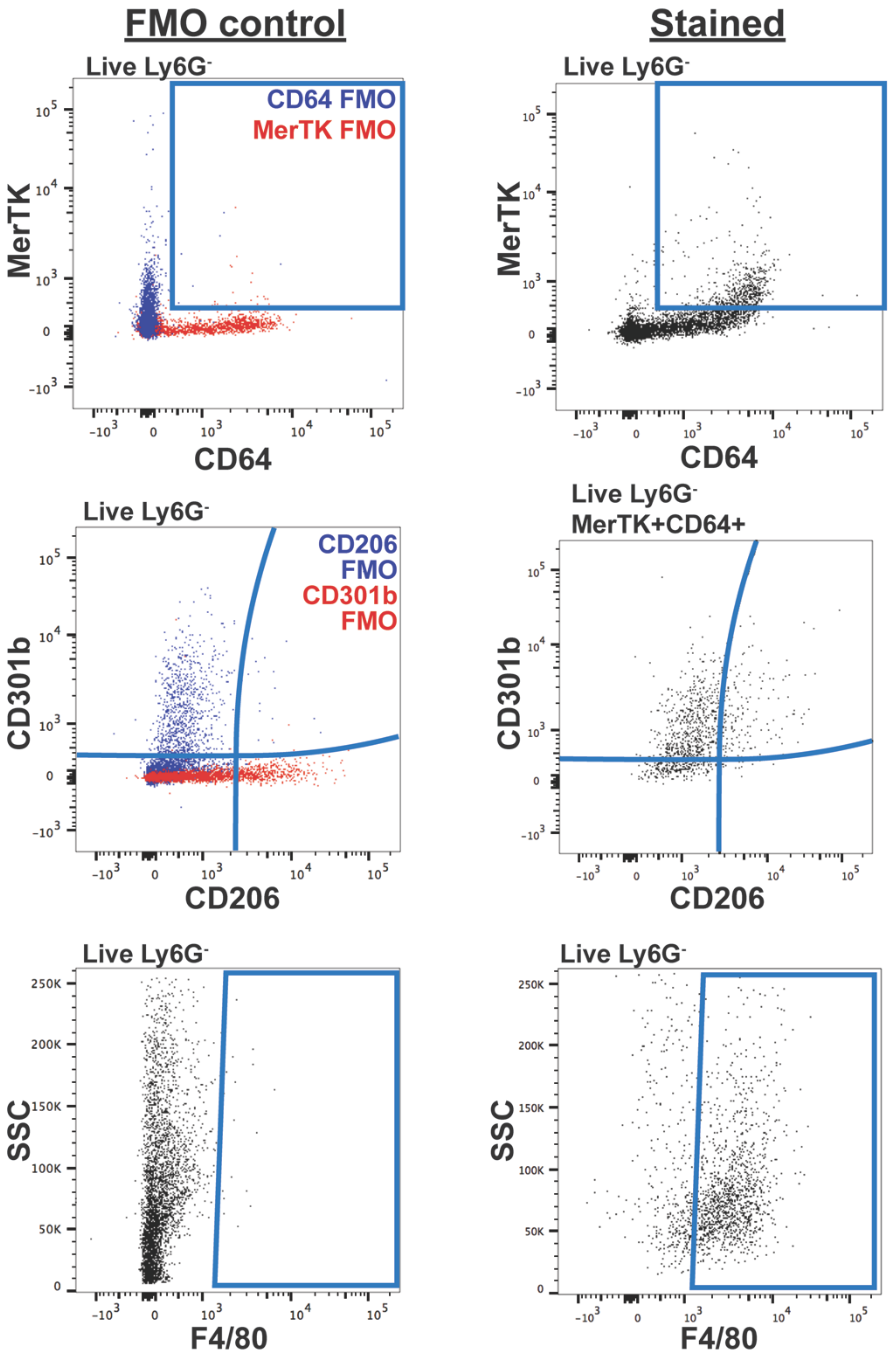

Supplemental Figure S3. Representative fluorescence minus one (FMO) gating. For MerTK, CD64, CD301b, CD206, and F4/80, gates were drawn based on the plots of the FMO samples. Positive cells were identified as cells falling above the fluorescence of the FMO control.

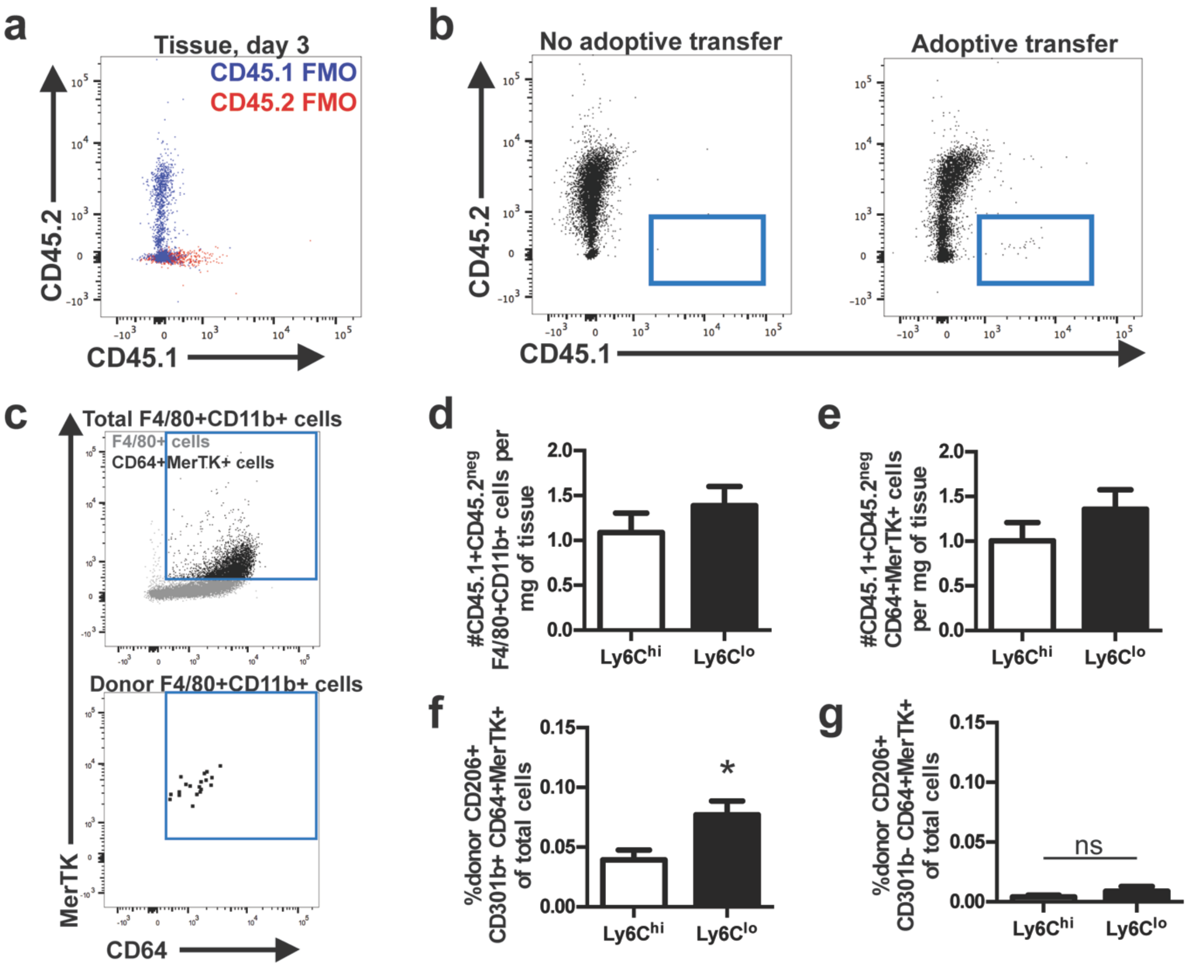

Supplemental Figure S4. (a) Ly6C^{hi} or Ly6C^{lo} monocytes from CD45.1 mice were adoptively transferred into CD45.2 recipient mice at the time of surgery. Fluorescence minus one (FMO) gating identifies CD45.1+CD45.2- donor-derived cells. **(b)** CD45.1+CD45.2- donor cells were detected in dorsal tissue digests 3 days post-injury only in animals that received adoptive transfer. **(c)** CD64+MerTK+ is a more discriminating macrophage population within F4/80+CD11b+ cells. 25.3 ± 2.1% of all F4/80+CD11b+ cells are CD64+MerTK+; however, nearly all donor-derived F4/80+ cells are CD64+MerTK+ (90.0 ± 3.8%). **(d)** No differences in the number of donor-derived F4/80+ or **(e)** CD64+MerTK+ macrophages per milligram of tissue were detected in mice receiving adoptive transfer Ly6C^{lo} monocytes compared to mice receiving Ly6C^{hi} monocytes. **(f)** A higher frequency of donor-derived CD301b+CD206+ macrophages (CD64+MerTK+) were detected in mice receiving adoptive transfer Ly6C^{lo} monocytes compared to mice receiving Ly6C^{hi} monocytes. **(g)** No differences in the frequency of donor-derived CD301b-CD206+ macrophages (CD64+MerTK+) were detected in mice receiving adoptive transfer Ly6C^{lo} monocytes compared to mice receiving Ly6C^{hi} monocytes. Data presented as mean ± S.E.M. Statistical analyses were performed using two-tailed t-tests *$p<0.05$, n=5-6 animals per group.

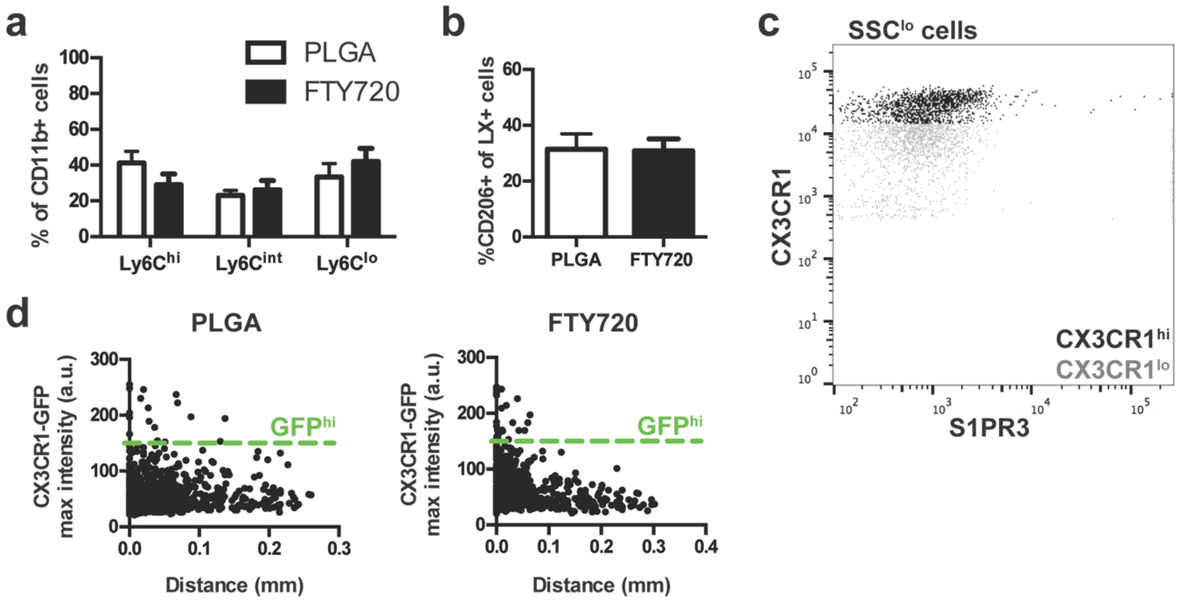

Supplemental Figure S5. (a) On-site delivery of FTY720 results in a modest increase in frequency of Ly6C^{lo} and Ly6C^{int} monocytes, but not Ly6C^{hi} monocytes in dorsal tissue at 3 days post-injury. **(b)** Local FTY720 delivery does not change the conversion efficiency of labeled circulation-derived Ly6C^{lo} monocytes to LX+CD206+F4/80+CD11b+ macrophages within dorsal tissue at 3 days post-injury. Data presented as mean ± S.E.M. n=10-11 animals per group. **(c)** Representative dot plot shows that blood-derived CX3CR1^{hi} monocytes are higher in S1PR3 expression than CX3CR1^{lo} monocytes. **(d)** DWCs were implanted on CX3CR1$^{GFP/+}$ transgenic mice and imaged intravitally at day 1 post-surgery. Plots show CX3CR1^{hi} cells clustered at shorter distances to vessels with FTY720 treatment.

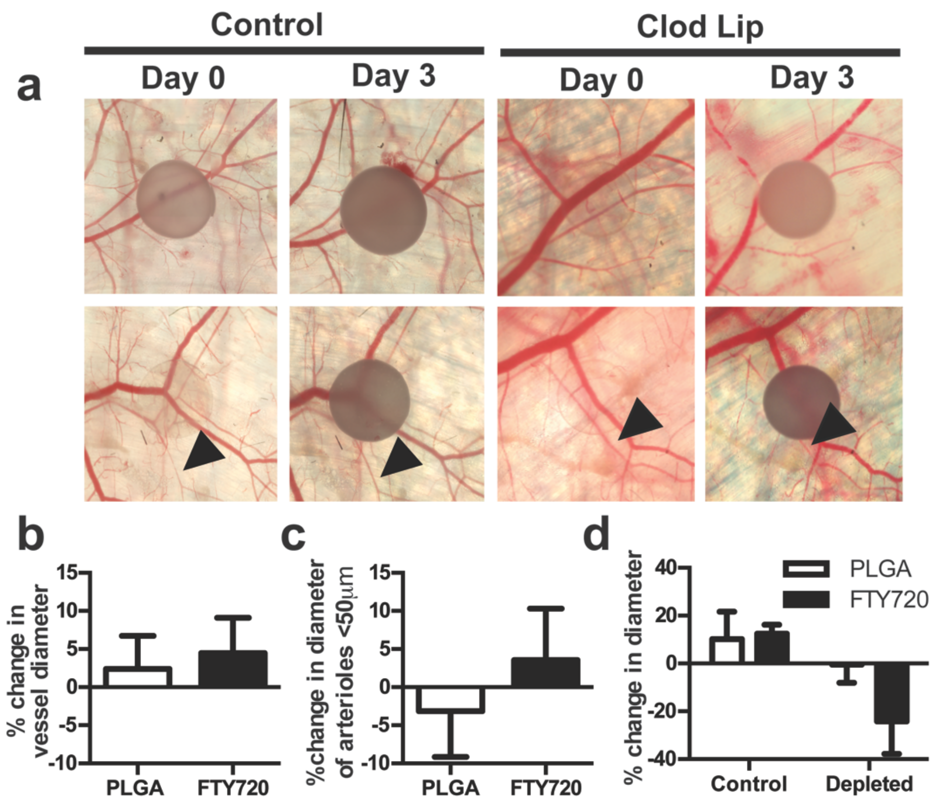

Supplemental Figure S6. **Recruitment of non-classical Ly6C^{lo} monocytes supports peri-implant arteriogenesis. (a)** Intravital brightfield images of window chambers containing PLGA control films or FTY720 films, 3 days post-surgery. Black arrows indicate arteriole expansion from day 1 – day 3 in the untreated FTY720 animals, and arteriolar regression from day 1 – day 3 in FTY720 animals treated with clodronate liposomes. **(b)** FTY720 induces minimal expansion of blood vessels at 3 days post-surgery; however, **(c)** a stronger effect is observed if measurement is limited to small arterioles less than 50μm in diameter. n=8-10. **(d)** Reduction of Ly6C^{lo} monocytes with clodronate liposomes reduces arteriogenesis. In the absence of Ly6C^{lo} monocytes, FTY720 inhibits arteriogenesis. n=4-10 animals per group. Data presented as mean ± S.E.M.

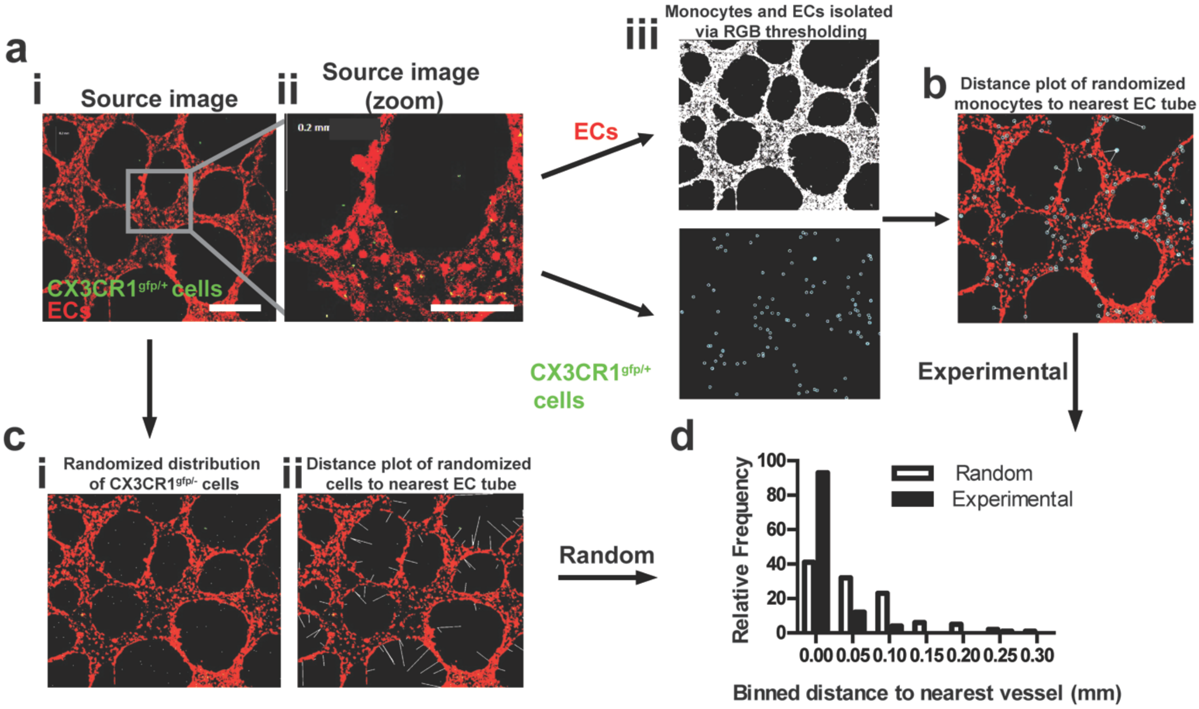

Supplemental Figure S7. Comparison of distance to endothelial cell networks of sorted CX3CR1$^{GFP/+}$ monocytes vs. randomized distribution. (a, i, ii) CX3CR1^{hi} cells were seeded within labeled endothelial cell tube networks and imaged. **(a, iii)** Monocytes and endothelial cell channels are separated and events isolated via RGB thresholding. **(b)** Monocyte to closest endothelial cell tube distance calculated within Matlab. **(c, i)** Identical number of cells as CX3CR1+ monocytes are randomly placed on endothelial cell image. **(c, ii)** The randomly placed cell to tube distance is calculated as in (b). **(d)** Comparison of randomly distributed and experimental CX3CR1^{hi} cells show that the experimental group has higher frequency of cells within 20μm of endothelial tube network.
